# Transcriptome changes in *Arabidopsis thaliana* infected with *Pseudomonas syringae* during drought recovery

**DOI:** 10.1038/s41598-017-09135-y

**Published:** 2017-08-22

**Authors:** Aarti Gupta, Muthappa Senthil-Kumar

**Affiliations:** National Institute of Plant Genome Research, Aruna Asaf Ali Marg, New Delhi, India

## Abstract

Field-grown plants experience cycles of drought stress and recovery due to variation in soil moisture status. Physiological, biochemical and transcriptome responses instigated by recovery are expected to be different from drought stress and non-stressed state. Such responses can further aid or antagonize the plant’s interaction with the pathogen. However, at molecular level, not much is known about plant-pathogen interaction during drought recovery. In the present study, we performed a microarray-based global transcriptome profiling and demonstrated the existence of unique transcriptional changes in *Arabidopsis thaliana* inoculated with *Pseudomonas syringae* pv. *tomato* DC3000 at the time of drought recovery (drought recovery pathogen, DRP) when compared to the individual drought (D) or pathogen (P) or drought recovery (DR). Furthermore, the comparison of DRP with D or DR and P transcriptome revealed the presence of a few common genes among three treatments. Notably, a gene encoding proline dehydrogenase (*AtProDH1*) was found to be commonly up-regulated under drought recovery (DR), DRP and P stresses. We also report an up-regulation of pyrroline-5-carboxylate biosynthesis pathway during recovery. We propose that *AtProDH1* influences the defense pathways during DRP. Altogether, this study provides insight into the understanding of defense responses that operate in pathogen-infected plants during drought recovery.

## Introduction

Plants challenged with a host pathogen often experience prolonged or episodic drought in field conditions. Episodic drought is characterized by dry-wet cycles. The host plant water status at the time of pathogen infection primarily decides the outcome of many foliar plant-pathogen interactions. This interaction also influences host plant growth apart from modulating defense pathways^1–5^. Previous reports have shown that upon re-watering, drought stressed plant undergoes a transition state which is different from that of both, drought stress and control conditions. Importantly, several physiological and molecular changes are known to occur during recovery^6–8^. These changes influence not only the plant vigor but also shape plant-pathogen interactions. For example, upon re-watering, plants re-open their stomata^9^ providing an opportunity to the foliar pathogen to colonize the once restricted mesophyll tissues. During drought stress, increased cell permeability leads to nutrient leakage into apoplast^10^ which may facilitate multiplication of some pathogens^11^. However, during recovery, the membrane permeability is normalized^12^, and plants channelize the nutrients to growth processes thereby limiting the nutrient availability to the invading pathogen.

In addition to these changes, several drought-induced physiological and transcriptomic changes in recovered plants have been reported to completely revert to non-stress levels^7, 9, 13^ which might also modulate plant-pathogen interaction. For instance, expression levels of drought-induced genes encoding for pyrroline-5-carboxylate synthase (AtP5CS), lipid transfer protein, protein phosphatase 2 C (PP2C), beta helix loop helix (bHLH) and late embryogenesis abundant (LEA) family proteins reverted to levels under control conditions after re-watering^7^. The increased concentrations of ABA under drought stress which contribute to plant susceptibility towards some pathogens^2^ are known to be completely recovered to uninduced levels after rehydration^7^. Thus, drought and recovery instigated physiological changes are likely to play a role in the plant-pathogen interaction.

In this regard, understanding the interaction of pathogen in plants recovering from drought is important. However, such studies were not widely reported at molecular level. In the present study, we have investigated the global transcriptome changes in drought-recovering *Arabidopsis thaliana* plants to *Pseudomonas syringae* pv. *tomato* DC3000 (Pst DC3000) infection. Our results indicate a difference in the transcriptome of drought recovering pathogen infected plant with respect to pathogen only treated plant. The genes involved in proline, polyamine metabolism and sugar transport were found to be specifically altered during this interaction. Altogether, we highlight how the defense pathways and primary metabolism are altered in infected plants during stress recovery.

## Materials and Methods

### Plant growth conditions

*Arabidopsis thaliana* ecotype Columbia-0 [Arabidopsis Biological Resource Center (ABRC), accession number CS70000] wild type and *atprodh1* (Salk_119334 C) and *atprodh2* (SALK_008533) mutant plants were grown and maintained as per the protocol recommended by ABRC. Briefly, seeds were sown at the density of one seed per 19 cm^2^ in pot with defined weight of pre-wet soil mix (3:1 vol/vol ratio of agropeat, Prakruthi Agro Tech, Bangalore, India and vermiculite, Keltech Energies Ltd, Maharashtra, India) and were stratified for 48 h in dark at 4 °C. Plants were grown under controlled short-day conditions (8 h light, 16 h dark) with 200 µE m^−2^ s^−1^ light intensity, 75% humidity, and 20 °C constant temperature in a growth chamber (PGR15, Conviron, Winnipeg, Canada). Plants were bottom irrigated alternatively with water or Hoagland solution (Cat # TS1094, Himedia Laboratories, Mumbai, India) every alternate day.

### Pathogen inoculum preparation

A single colony of *Pseudomonas syringae* pv. *tomato* DC3000 (Pst DC3000) was inoculated in King’s B liquid medium (Cat # M1544, Hi-media Laboratories, Mumbai, India) supplemented with rifampicin (50 μg/mL). The bacteria was grown at 28 °C on a rotary shaker at 200 revolutions per minute (rpm) for 12 h until the optical density at 600 nm (OD_600_) reached 0.4. The cells were centrifuged at 4270 g for 10 min followed by washing with sterile water thrice. The resultant pellet was re-suspended at inoculum concentrations of 5 × 10^3^ and 1 × 10^4^ colony forming units (CFU)/mL in sterile water.

The bacterial suspension at the defined concentration was inoculated on the abaxial side of the leaves (37-d-old plant) by needleless syringe. Subsequently *in planta* bacterial number was assessed from the leaves at 0, 24 and 72 hour post inoculation.

### Drought stress imposition

*A*. *thaliana* plants were grown (32-d-old) in fully saturated soil (10 g dried potting mix) with 100% soil moisture content (field capacity, FC, Ψw = −2.89 MPa) till the start of the stress experiments. The gravimetric method was followed for drought stress imposition^14^. Drought stress was commenced by withholding irrigation until the potted plants reached 40% FC (Ψw = −3.9 MPa). The drought stressed plants were maintained at 40% FC until the end of the experiment (four days post treatment, dpt) by replacing the amount of water lost through evapotranspiration. Control plants were maintained at 100% FC throughout the experiment.

### Drought recovery treatment

A batch of drought-stressed plants (37-d-old) at 40% FC were bottom irrigated till the pot mix is saturated (100% FC) and were maintained at 100% FC until the end of the experiment. Leaf samples were harvested and were used for the RT-qPCR experiment.

### Pathogen inoculation on re-watered plants

A batch of drought stressed plants were exposed to pathogen infection during recovery (DRP). For this, plants (37-d-old) that have reached 40% FC were bottom irrigated till the pot mix is saturated (100% FC). Two hours after re-watering, pathogen inoculum (5 × 10^3^ for transcriptome analysis and 1 × 10^4^ CFU/mL for physiological assays) was syringe infiltrated through the abaxial side of the leaves. These plants recovering from drought stress coupled with progressive pathogen multiplication were maintained at 100% FC until the end of the experiment (4 dpt).

Leaf samples were then taken for physiological assays and microarray experiment. The data acquired from the physiological and molecular studies under D and DR treatments was normalized with well-watered (absolute control), and P and DRP treatment with mock (water only) infiltrated plants. The outline of DRP treatment is provided in Supplementary Fig. S1. Pathogen inoculation on drought-stressed plants (DP) was performed according to the previous report^15^.

### Total RNA isolation

Total RNA was extracted from 100 mg leaf tissue with RNeasy Plant Mini Kit (Cat # 74904, Qiagen, Hilden, Germany) as per manufacturer’s guidelines. Leaf tissue from the third tier of rosette was harvested, and leaves from three different plants were pooled for each biological replicate. For each treatment, two biological replicates were sampled. The pooled sample was homogenized in liquid nitrogen into a fine powder, and the total RNA was extracted. Following total RNA isolation, DNase digestion (Cat # 79254 RNeasy/QlAamp columns, Qiagen, Hilden, Germany) was performed to remove contaminating DNA, according to the manufacturer’s instructions. RNA integrity was analyzed using the Agilent 2100 Bioanalyzer (Agilent Technologies, California, USA) with RNA 6000 Nano Chips (Cat # 5067-1511, Agilent Technologies, California, USA), following the manufacturer’s protocol. RNA integrity numbers (RIN) ranging from 7.0 to 7.5 were used for the microarray experiment.

### Microarray labeling and hybridization

cDNA synthesis, dye labeling, microarray hybridization, and scanning were performed as per the protocol provided by the manufacturer (Affymetrix, California, USA). Briefly, microarray experiment was conducted using Whole Transcript (WT) Expression Arrays (Cat # 902281, Affymetrix, California, USA). Total RNA (500 ng, RIN = 7.0 to 7.5) was isolated from leaf tissue sampled at 24 hpt and was reverse transcribed to synthesize single-stranded cDNA with T7 promoter sequence at 5′ end. Double-stranded cDNA was prepared from template cDNA while degrading the residual RNA at the same time. *In vitro* transcription (IVT) of the second-stranded cDNA template was carried out to prepare complementary RNA (cRNA) using T7 RNA polymerase. cRNA was reverse transcribed to make sense strand cDNA containing dUTP (at a fixed ratio relative to dTTP). Template RNA was removed following RNase H mediated hydrolysis. Sense-strand cDNA was purified, fragmented at the dUTP residues and labeled using the biotin linked-DNA labeling reagent. Biotin-labeled single stranded (ss)-cDNA was mixed with hybridization master mix and loaded onto cartridge (GeneChip Gene 1.0 ST, Cat # 901915, Affymetrix, California, USA). Hybridization was carried out for 16 h at 45 °C, and 60 rpm followed by washing, staining, and scanning using GeneChip hybridization, wash, and stain kit (Cat # 900720, Affymetrix, California, USA). The microarray experiment was performed keeping two biological replicates for each treatment^16^.

### Microarray data processing and analysis

Scanned image files (.CEL) were imported into Expression Console (EC, Affymetrix). The microarray data were normalized using RMA algorithm (Expression Console) and converted into the.chp format. EC processed files were imported into Transcriptome Analysis Console, and one way-ANOVA was employed to obtain differentially expressed genes between treatment and control condition. Differentially expressed genes (≥2-fold) between two conditions (treatment over control) with *p* value ≤ 0.05 were selected for further analysis. The selected features were categorized such that positive values for up- and negative values for down-re﻿gulated gen﻿es were designated.

Functional categorization of genes was done as per Gene Ontology (GO) biological and molecular function according to TAIR 10. Differentially expressed genes among different treatments were compared using Venn diagrams (Venny 2.0). Based on Affymetrix probe IDs, lists of DEGs were compared using Venn diagram and genes were regarded as unique to combined DRP treatment (or individual stress treatments) and common between individual and combined treatments.

Significantly over-represented GO terms associated with differentially regulated gene lists were determined using ‘The Biological Networks Gene Ontology’ (BiNGO) tool^17^. The enrichment score was calculated for GO processes, and *p* values were corrected using the Bonferroni algorithm for minimizing multiple testing errors, and the *p *value threshold was set at <0.05.

The pathway networks were determined by the input of selected gene lists into KEGG Automatic Annotation Server (KAAS, http://www.genome.jp/tools/kaas/) and MAPMAN (http://mapman.gabipd.org/web/guest/mapman). Heat maps were drawn with fold change values using GENE-E software (http://www.broadinstitute.org/cancer/software/GENE-E/).

### Validation of transcriptomic data

The expression profile of the 13 selected genes in response to DRP stress and 52 genes common among DR, P and DRP stress as observed from microarray data was validated using the real-time quantitative PCR (RT-qPCR). Raw data for the figures are provided in Supplementary Files S1, S2 and S3. For the purpose, total RNA was isolated using Trizol^TM^ reagent (Cat # 15596018, Thermo Fisher Scientific, Massachusetts, USA) as per the protocol provided by the manufacturer. Gene-specific primers were designed using Primer 3 software^18^. The details of primers used in the study are provided in Supplementary File S4. Total template cDNA was prepared from 5 μg of total RNA in a reaction volume of 50 μL using Verso cDNA synthesis kit (Cat # AB1453A, Thermo Fisher Scientific, Massachusetts, USA). Reaction mix contained 1 µL of template cDNA (5- fold diluted), 750 nM of gene specific primers and 5 µL of Brilliant III Ultra-Fast SYBR Green QPCR master mix (Cat # 600882, Agilent Technologies, California, USA) in a final volume of 10 µL. The reaction was carried out in an ABI Prism 7000 sequence detection system (Applied Biosystems, California, USA). Ct values obtained for target gene were normalized with Ct values obtained for *AtACTIN2* (AT3G18780) gene. Fold change in gene expression in stressed samples was quantified using comparative D cycle threshold (CT) method relative to the non-stressed control samples^19^. Data from three to four independent biological replicates were used to interpret the results.

### *In planta* bacterial count

To count the bacterial numbers in leaves, circular discs measuring 0.785 cm^2^ were cut and treated with 0.01% H_2_O_2_ for 20 sec to remove epiphytic microbial population. Each leaf disc was well ground in 1000 µL of sterile water, serially diluted in sterile water and plated on King’s B agar medium containing 50 mg/L of rifampicin. Bacterial numbers were determined using the following formula^20^ (equation 1).

1 $${\rm{Bacterial}}\,{\rm{multiplication}}\,({\text{CFU}/\text{cm}}^{2})=\frac{\frac{{\rm{Number}}\,{\rm{of}}\,{\rm{colonies}}\times {\rm{volume}}\,{\rm{of}}\,{\rm{homogenate}}\,(\mu L)\times {\rm{dilution}}\,{\rm{factor}}}{{\rm{volume}}\,{\rm{plated}}}}{{\rm{Leaf}}\,{\rm{area}}\,({{\rm{cm}}}^{2})}$$

### Relative water content

To assess the tissue water status, relative water content (RWC) of the leaf (6 cm^2^) was measured as described by Flower and Ludlow^21^. Fresh weight (FW) was estimated immediately after harvesting the samples, and subsequently, the samples were floated on de-ionized water at 22 °C. The surface moisture was removed by blotting gently with filter paper and turgid weight (TW) was noted. Samples were then oven dried at 60 °C for 72 h and dry weight (DW) was obtained. RWC was calculated using the following formula (equation 2):

2 $${\rm{RWC}}( \% )=\frac{{\rm{FW}}-{\rm{DW}}}{{\rm{TW}}-{\rm{DW}}}\times 100$$

### Electrolyte leakage assay

Leaf discs (0.785 cm^2^) were excised from the third tier of the rosette (same developmental stage). Discs were then rinsed in deionized water for 2 min to remove cut end leachates. Two discs per sample were gently agitated (at 60 rpm) in 20 mL of deionized water (1.3 μS/cm electrical conductivity) for 12 h at 20 °C. Minimum of six biological replicates were used for each treatment. Electrical conductance of the leachates in the bathing solution was measured using a conductivity meter (Model-1602, EC-TDS-SAL Meter, Esico International, Parwanoo, India). Leaf discs suspended in the bathing solution were autoclaved to release 100% electrolytes in the same bathing solution, and conductivity was measured again. Electrolyte leakage was calculated by taking ratios of the initial conductivity reading to the reading after autoclaving^22^.

### Proline estimation

Leaf sample was harvested at 24 hpt, frozen in −80 °C and was ground in 1 mL (3%) sulfosalicylic acid. Acid ninhydrin reaction was pursued, and free proline was extracted in toluene. Colorimetric estimation of proline was carried out at 520 nm as described by Senthil-Kumar and Mysore^23^, except deduction of P5C.

Proline content was measured using the following formula (equation 3):

3 $${\rm{Proline}}({\rm{\mu }}g/g\,{\rm{dry}}\,{\rm{weight}})=\frac{{{\rm{OD}}}_{520}\,{\rm{of}}\,{\rm{the}}\,{\rm{sample}}\times {\rm{Volume}}\,{\rm{of}}\,{\rm{the}}\,\text{extract}(\text{mL})\times {\rm{factor}}}{{\rm{Volume}}\,{\rm{of}}\,{\rm{the}}\,{\rm{aliquot}}\,({\rm{mL}})\times {\rm{Weight}}\,{\rm{of}}\,{\rm{the}}\,{\rm{tissue}}\,({\rm{mg}})}$$

### Cell death assay

Leaf samples were harvested at three days post treatment (dpt), and cell death was assayed as described by Koch and Slusarenko^24^. Leaves were stained with trypan blue solution (0.02 g trypan blue, 8% phenol, 8% glycerol, 8% lactic acid, 8% water, 62% absolute alcohol) for 6 h and were subsequently destained for overnight in chloral hydrate solution. The stained leaves were observed under a bright field microscope. The images were captured using a digital camera (Nikon Digital Sight DS-Rs1) mounted on a Nikon Stereo zoom AZ100 Microscope. The extent of cell death was measured as intensity using ImageJ software (http://imagej.nih.gov/ij/).

### Statistical analysis

Data presented in bars are an average of biological replicates with error bars marking ± SEM. The numbers of biological replicates considered for each experiment are mentioned for each figure in their legend. Data presented in Supplementary Fig. S2 from biochemical and physiological assays were subjected to one-way ANOVA, and significant differences between treatments were determined using *post-hoc* Tukey’s pairwise comparison test at the 5% confidence level (SigmaPlot 11.0, Systat Software Inc., California, USA).

## Results and Discussion

### Physiological responses of *A*. *thaliana* under DRP treatment

*Arabidopsis thaliana* was exposed to individual drought (D) and pathogen (P) stresses and combined treatment of pathogen inoculation at the time of rehydration (drought recovery pathogen, DRP) (Supplementary Fig. S1A). The impact of water withholding on plants was assessed by estimating the relative water content (RWC) of the leaf. The plants at 40% FC showed ~55% RWC as compared to the 90% RWC in turgid leaves of control plants at 100% FC. When compared to control plants, pathogen infiltration did not lead to a change in leaf RWC in P stressed plants. However, DRP stressed leaves showed 74.6% RWC (Supplementary Fig. S2A). Both soil and leaf water status measurements indicated gradual saturation of soil moisture content and an increase in RWC under DRP treatment as compared to D stressed plants (Supplementary Fig. S1B). Pathogen was inoculated for P (2.4 Log CFU/mL) and DRP (2.8 Log CFU/cm^2^) treatment. After 24 h of *in planta* multiplication, the bacterial number increased to 6.7 and 5.8 Log (CFU/cm^2^) in P and DRP plants, respectively (Supplementary Fig. S2B). This indicates a reduced *in planta* bacterial multiplication in DRP stressed plants when compared to P only plants (Supplementary Fig. S2D). In addition, overall, observations on RWC and *in planta* bacterial number from both individual and combined DRP plants indicated successful drought stress imposition and pathogen infection, respectively.

The stress inflicted alterations in membrane permeability leading to solute leakage^10, 25–27^ are usually restored during drought recovery^12^. With this notion, we assessed the membrane leakage and showed that the leakage in D and P stressed plants were 26% and ~22%, respectively, when compared to control plants (Supplementary Fig. S2C). DRP stressed plants showed ~30% leakage over control plants. This was not significant when compared to D stressed plants, but was higher in comparison to the P stressed plants. The results suggest that the high electrical conductivity values in DRP plants could be due to drought-induced solute leakage which might be retained in the apoplast during recovery in addition to leakage caused by the pathogen (Supplementary Fig. S2C). Our results also indicate that the drought-recovery treatment did not amount to an additional stress to the DRP plants when compared to D or P stressed plants (Supplementary Fig. S2D).

### Distinct transcriptome changes in combined DRP plants compared to individual DR, D and P treated plants

To further delineate the molecular responses in DRP plants, we performed leaf transcriptome analysis by microarray under drought, pathogen and DRP treatments. The microarray data were submitted to Gene Expression Omnibus (GEO, GSE79681). Data were analyzed using Expression Console (EC, Affymetrix) and with ANOVA *p* value cut-off set at less than 0.05. Expression values of the transcripts under different conditions were compared and clustered (hierarchal clustering, Supplementary Fig. S1C). Hierarchal clustering showed that different controls viz., mock infiltrated (M), and absolute control conditions were clustered closely, and pathogen stress was close to DRP treatment (Supplementary Fig. S1C).

Using Transcriptome Analysis Console (TAC, Affymetrix), genes holding significant expression in each stress condition (ANOVA *p* value < 0.05) were compared to controls, and differentially expressed genes (DEGs) that have more than 2-fold change cut off were shortlisted (Supplementary Files S1, S2 and S3). The numbers of statistically significant differentially expressed transcripts under each stress condition are presented in Supplementary File S1. Following drought, pathogen and combined DRP stress treatments, we noted 1166, 1084 and 553 numbers of differentially expressed transcripts. Results drawn from transcriptome analysis in DRP treated plants revealed the presence of genes associated with drought, pathogen and drought-recovery. Genes encoding proline dehydrogenase (AtProDH1), dehydration response element-binding protein (AtDREB2A), toll-interleukin resistance (TIR, AT1G72920) and WRKY33 were most up-regulated while those encoding heat shock protein (HSP17, AT5G12020) and pathogen related protein (AtPR1) were down-regulated under DRP treatment (Supplementary Fig. S3). Furthermore, the transcriptome data showed that the recovery specific genes; *AtProDH1* and drought repressed 4 (*DR4*) were up-regulated under DRP stress^28, 29^. *RD29B* (a well-known drought-induced gene) expression was reduced in DRP while its expression was 13-fold higher in drought stressed plants than control (Supplementary File S1). From our results on physiological and transcriptomic studies, we infer that drought recovery is a transition state (between drought and control), thereby both drought stress specific and drought reversed traits along with some pathogen-specific transcriptomic changes prevails in DRP stressed plants.

**Figure 1:**
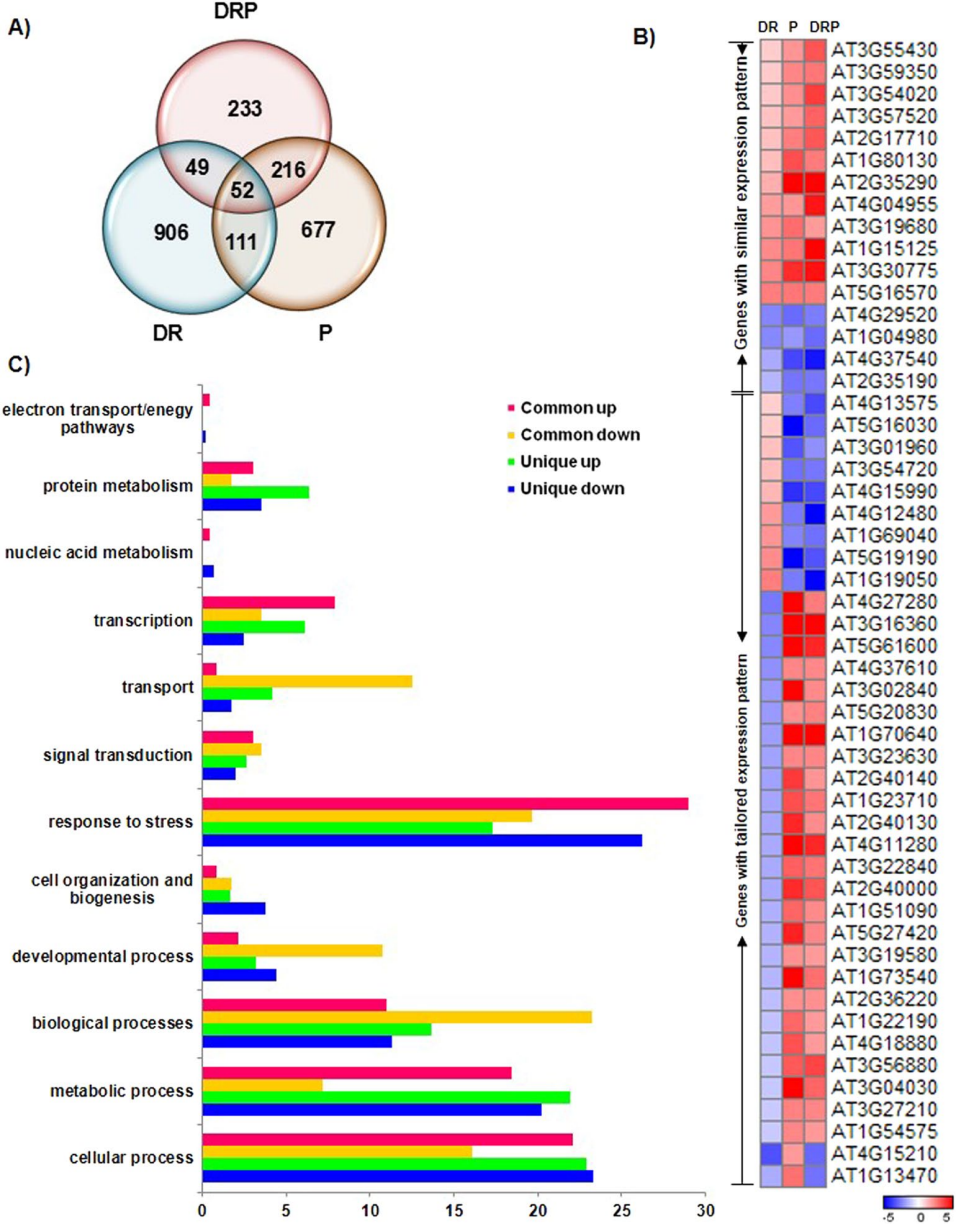
Overview of transcriptome profile of *Arabidopsis thaliana* exposed to drought-recovery-pathogen treatment in comparison to drought recovery and pathogen treatments. *A*. *thaliana* was exposed to *Pseudomonas syringae* pv. tomato DC3000 (Pst DC3000; P) and combined drought-recovery-pathogen (DRP) treatments as outlined in Figure S1. Microarray hybridization on Affymetrix WT gene chip array was conducted using total RNA isolated from leaf samples (38-d-old plants) harvested at 24 hours post treatment (hpt). Differentially expressed genes (DEGs) in each stress treatment were identified in comparison to control conditions and threshold was set at change greater than 2 fold and ANOVA *p* value < 0.05. Differentially expressed genes under drought recovery (DR) were retrieved from Coolen *et al*.^30^. Venn diagram between DEGs in individual (DR and P) and combined (DRP) stress revealed the presence of transcripts exclusively under DRP stress and were regarded as ‘unique’ genes, and ‘common’ transcripts shared between individual and combined stresses (**A**). Expression profile of genes common among DR, P and DRP is presented in the form of heat map. Common transcripts were categorized as genes with similar expression pattern in all the three stress conditions and as genes with ‘tailored’ expression pattern under different stresses (up-regulated vs. down-regulated and vice versa) (**B**). Expression values were used to plot heat maps using GENE-E software (http://www.broadinstitute.org/cancer/software/GENE-E/). Colour bar scale shows the fold change range with a red and blue colour representing up- and down-regulation respectively. Corresponding gene names and descriptions for the gene IDs presented in the figure are provided in Supplementary File S1. Genes common to individual and combined stress and genes unique to the DRP stress were functionally categorized and represented based on gene ontology (GO) biological process (**C**).

In our present study, we also compared previously reported transcriptome changes during recovery from drought stress (DR)^6, 29, 30^. Differentially expressed genes under DR were retrieved from other close studies by Coolen *et al*.^30^ (transcriptome analysis at 24 h post recovery as in DRP) and Oono *et al*.^6^ During the analysis, we found consistency between the expression pattern of recovery inducible genes between DR and DRP treatment (Fig. 1, Supplementary Fig. S4). Through Venn intersections, we observed the existence of 223 transcripts unique to DRP and 52 transcripts common among combined DRP and individual DR and P stresses (Fig. 1A, Supplementary Fig. S4). Out of the 52 transcripts common, 16 genes exhibited similar expression pattern among different stresses, and other 36 were ‘tailored’ as indicated by the different expression pattern in all the three conditions (Fig. 1, Supplementary Fig. S4). The common genes with similar up-regulated expression include genes encoding proline dehydrogenase 1 (AT3G30775, AtProDH1), and putative galactinol–sucrose galactosyltransferase 2 (AT3G57520). Furthermore, the genes with similar down-regulated expression include ethylene-responsive transcription factor (AT1G22190, AtERF058), VQ motif-containing protein (AT3G56880) and Calcium-binding EF-hand family protein (AT4G27280) (Supplementary File S1). These genes were also found to exhibit similar expression in another DR study (Oono *et al*.^6^); Supplementary Fig. S4). To rule out the expression bias arising due to different experimental conditions in these studies, we validated the expression of 52 genes common among DR, P and DRP stressed conditions through RT-qPCR (Supplementary Fig. S5, Supplementary File S2). The RT-qPCR based validation of these genes under DR, P and DRP treatments also authenticated our microarray data. Altogether the presence of unique and common molecular signatures in DRP suggests that the DRP treatment is different from either of the individual DR or P only treatments.

To understand the molecular responses of DRP plants over individual D and P stresses, DEGs under D, P and DRP treatments were compared using Venn intersections (Supplementary Fig. S6A). The results revealed 227 unique genes in DRP transcriptome. Only 58 genes were commonly expressed among D, P and DRP stresses. Out of these, 29 genes exhibited similar expression pattern and other 29 exhibited tailored expression pattern in all the three stress conditions (Supplementary Fig. S6B). Genes with similar expression were those encoding for ubiquitin protein ligase (ATL31, AT5G27420), calmodulin binding proteins (CML24, AT5G37770), sugar transporter (AtSWEET4, AT3G28007), WRKYs (WRKY40, WRKY53; AT1G80840, AT4G23810), NAC036 (AT2G17040), and heat shock proteins (AT3G07770, AT2G04030) (Supplementary Fig. S6B). These genes have been reported to take part in the basal defense responses and also mediate the cross-talk among different abiotic and biotic stress responses^31^. For example, cytosolic calcium levels increase in response to both pathogen infection and water stress; this activates signaling cascades by calcium-interacting proteins such as Ca^2+^-dependent protein kinases (CDPKs), calmodulin and calcineurin B like proteins (CBLs). These signaling cascades are subsequently mediated by transcription factors, WRKY and NAC, and are culminated in cellular responses such as ubiquitin-mediated degradation pathways. Conclusively, we infer that these genes are involved in mediating crosstalk between D and P stress signals in DRP plants. Furthermore, the genes with tailored expression were coding for tyrosine aminotransferase (AT2G24850), and LURP1 (AT2G14560). These genes were down-regulated under both D and DRP but were up-regulated under P stress. In addition, the genes encoding heat shock proteins (AT3G07770 and AT2G04030) and two component response regulator ARR7 (AT1G19050) were down-regulated during P and DRP but were up-regulated during D stress (Supplementary Fig. S6B). Collectively, the expression pattern of common genes among D, P and DRP transcriptome showed that the DRP transcriptome bears close resemblance with P stressed transcriptome. The reflected differences in D and DRP are specific to recovery^6, 7, 28^.

In addition, we found 56 genes that overlap between D and DRP stress where 26 and 30 genes showed similar and tailored expression, respectively, in DRP when compared to D (Supplementary Fig. S7A). The expression pattern of *AtPR1* and *AtPR5* genes were commonly down-regulated, and those encoding TIR-NB-LRR disease resistance protein (AT1G56510) and PP2C3 (AT1G07430) were up-regulated in D and DRP stress transcriptome, indicating the continuance of some of the drought-induced events during recovery. On the contrary, among the tailored genes, those genes coding for hydroxyproline-rich glycoprotein family proteins (AT1G23040 and AT5G49280), proline-rich family proteins (AT5G12880), GDSL esterase/lipase (AT1G28660) and beta-D-xylosidase 4 (AT5G64570) that showed down-regulation under D stress condition were up-regulated under DRP stress (Supplementary Fig. S7A, Supplementary File S2). A number of tailored genes exhibited a reverse expression pattern under DRP stress in comparison to D condition indicated a recovery state, a result also observed in a previous study^6^. For instance, a few genes which were induced in response to DRP treatment exhibited a down-regulated expression during D stress (Supplementary Fig. S8). The presence of common genes with similar and tailored expression in DRP and D stressed plants indicated a transition state (between drought and control) of these plants.

We also observed 212 genes that were commonly expressed in DRP and P stresses, wherein 208 genes showed similar expression and only four genes showed tailored expression (Supplementary Fig. S7B). For example, one batch of genes with tailored expression pattern were those encoding beta amylase 5 (*AtBAM5*), WRKY DNA binding protein 54 (*AtWRKY54*) and short chain dehydrogenase reductase 3a (*AtSDR3*) that were up-regulated under P only stress but down-regulated under DRP stress (Supplementary File S2). Prevalence of similar responses between P and DRP plants showed a close association between that of DRP with P induced transcriptome responses. These results were in concurrence with hierarchal clustering data (Supplementary Fig. S1C). Recently, Olivas *et al*.^32^ imposed sequential stress on *A*. *thaliana* with prior drought stress followed by recovery and *Botrytis cinerea* or *Pieris rapae* infection and inferred that the effect of a previous stress on plant transcriptome is abrogated by the subsequent stress imposed.

**Figure 2:**
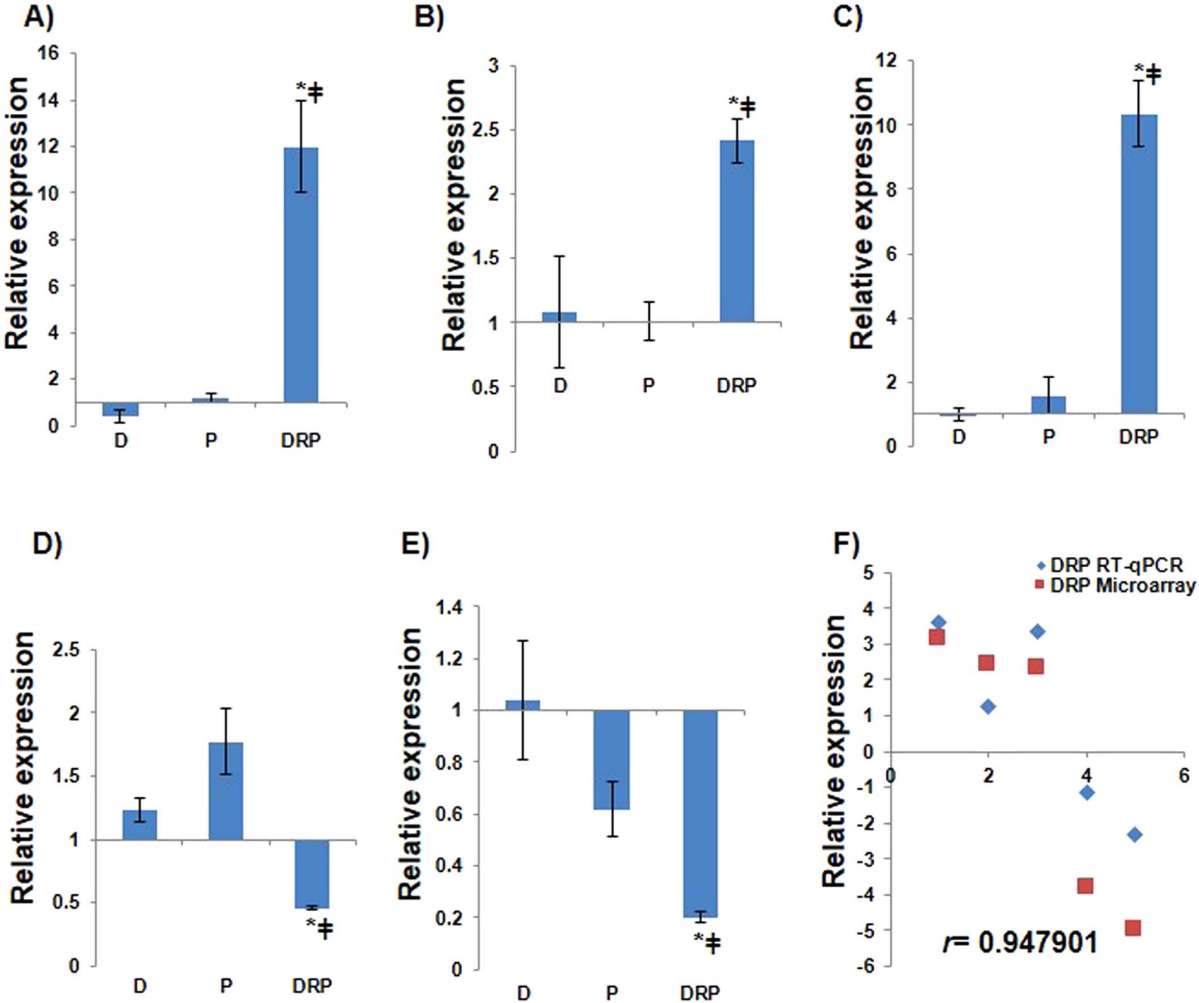
RT-qPCR validation of microarray data from DRP treated plants. Highly expressed unique genes under DRP stress from microarray were selected and corresponding transcript accumulation under D, P and DRP stress treatment were compared by RT-qPCR analysis. Fold change in expression levels relative to the control samples were normalized to *AtACTIN2* gene expression. RT-qPCR based quantification was performed with three biological replicates (two technical replicates each). Graph represents the relative fold change values (compared to control or mock treatment) in the expression of genes encoding for AtACS11 (**A**) AtAPK4 (**B**) AT1G69570, a DOF-type Zinc finger type protein (**C**) AT3G49340, a cysteine proteinases superfamily protein (**D**) and AT5G44570, an unknown protein (**E**). Represented data are the average of three biological replicates from one experiment, and error bars show standard error of the mean (SEM). Significance was calculated using Student’s *t*-test where * and ^‡^ symbol shows significance at p < 0.05 over drought and pathogen stress respectively. Fold change values in gene expression obtained from microarray and RT-qPCR analysis under DRP stress treatments were compared with scatter plot (**F**). Gene names and descriptions for the gene IDs presented in the figure are provided in Supplementary File S2. Details of primers used in the study are provided in Supplementary File S4.

Besides shared genes, DRP transcriptome had genes with expression unique to DRP. These genes include those encoding NAC (NAC3 and NAC6), bZIP transcription factor (AT4G35900), PP2A (AT5G28900), serine/threonine protein kinase (SRKC2), lipid transfer protein (AT4G12500), cysteine proteinase (AT3G49340) and pathogenesis related protein 4 (PR4). RT-qPCR results for few genes unique to DRP showed expression pattern similar to the microarray, and this confirmed the unique expression of these genes under combined DRP treatment (Fig. 2, Supplementary Fig. S9). Taken together, these results concede to the existence of new state of stress in DRP plants that are different from that of individual stress. Also, the present observation is consistent with the observations of the previous studies on drought-pathogen interaction^3, 4, 15^.

To understand defense mechanisms under pathogen infection during drought (DP) and drought-recovery (DRP), DEGs under DRP stress were compared with those under DP stress condition (Supplementary Fig. S10). The expression profile of genes in both combined stress conditions was classified in three modes based on clustering pattern (Supplementary Fig. S10). Those clustered with a similar trend of expression pattern under DRP and DP stress were referred as ‘DRP-DP similar’. Similarly, those clustered with high expression under DRP stress in comparison to the DP stress were ‘DRP-DP pronounced’ and cluster with unperturbed (no change in expression) or opposite (up and/or down-regulation) expression under DP compared to DRP stress were referred as ‘DRP specific’. A larger fraction of genes belonged to ‘DRP specific class followed by ‘DRP-DP pronounced’ and ‘DRP-DP similar’ classes. This analysis indicates that the plant perceives DRP as altogether a different treatment when compared to DP stress. Furthermore, GO ‘response to stress’ was more prevalent in ‘DRP-DP similar’ and ‘DRP specific’ (over ‘DRP-DP pronounced’) implicating an existence of stress memory and uniqueness of DRP treatment. Developmental processes and cell organization and biogenesis were more prevalent in ‘DRP specific’, and these were absent from DP stress indicating the resumption of normal growth processes upon recovery in DRP (Supplementary Fig. S10). GO’transcription’ was down-regulated in all three classes. Electron transport and energy pathways were preferentially up-regulated in ‘DRP-DP pronounced’ responses. Essentially, in DRP stressed plants ‘DRP-DP similar’ responses were dominated, and this could be one of the reasons for the reduction in bacterial number.

### Global transcriptomic analysis revealed activated defense responses in DRP over D and P stressed plants

**Figure 3:**
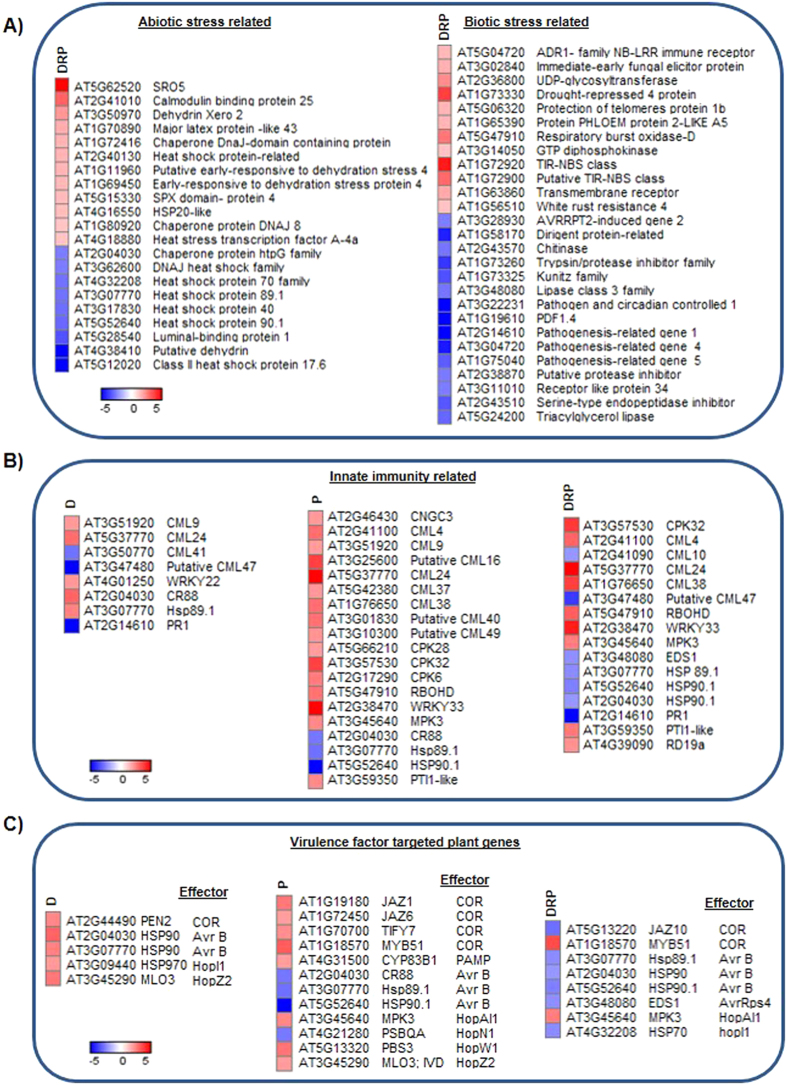
Expression profile of ‘defense’ and other ‘stress’ related transcripts under DRP treatment. DEGs (fold change cut-off ≥2) under individual D, P stresses and combined DRP treatment were associated with ‘defense’ and ‘stress’ related processes. Heat maps represent expression profile of stress-related genes (**A**). Differentially expressed genes were mapped onto abiotic and biotic stress category as provided by MAPMAN. Heat map represents expression of biotic stress related genes specifically involved in pathogen-associated molecular pattern (PAMP) triggered immunity (PTI) and effector-triggered immunity (ETI) pathways under individual drought (D), pathogen (P) and combined DRP treatment (**B**). Genes involved in PTI and ETI were curated from KEGG pathways. Heat map depicts expression pattern of biotic stress-related plant genes targeted by bacterial virulence factors including coronatine (COR) and PAMP or different bacterial effectors (secreted into the plant cell and contributing to the virulence) (**C**). The plant genes targeted by virulence factors were manually curated. Gene expression values were used to draw heat maps where colour bar scale shows the fold change range with a red and blue colour representing up- and down-regulation respectively. Gene IDs for the corresponding gene names presented in the figure are provided in Supplementary File S2.

Functional categorization of D, P and DRP stress transcriptome revealed not only enriched stress responses to drought stress and defense responses but also revealed many activated genes related to other stresses namely, cold, salt, oxidative stress, high light intensity, high temperature, wounding and herbivory (Supplementary Figs S11 and S12, Supplementary File S3). This data and earlier literature information on these stresses^7, 33^ suggest a probable activation of basal stress responses in D, P and DRP plants. In order to further dissect these ‘common’ molecular events, we organized DEGs under DRP treatment into stress wise categories and mapped them to different pathways. DRP stressed transcriptome exhibited up-regulation of abiotic stress related genes encoding calmodulin binding protein 25 (*AtCAMBP25*), dehydrin Xero2 (*AtLTI30*), early-responsive to dehydration stress protein (*AtERD4*), chaperone protein dnaJ 8 (*AtTOC12*), major latex protein like 43 (*AtMLP43*) and down-regulation of genes coding for heat shock proteins (AT4G32208, AT3G07770, AT3G17830, AT5G52640 & AT5G12020) (Fig. 3A). Biotic stress related genes viz., protection of telomeres protein 1b (*AtPOT1b*), UDP-glycosyltransferase 73C5 (*AtDOGT1*), protein phloem protein 2-LIKE A5 (*AtPP2*-A5), *AtDR4*, respiratory burst oxidase-D (*AtRBOHD*), TIR-NBS (AT1G72920) and TIR-NBS-LRR class (AT1G72900) were up-regulated while pathogenesis related proteins *AtPR1*, *AtPR4*, *AtPR5*, pathogen and circadian controlled 1 (*AtPCC1*) were down-regulated in DRP plants (Fig. 3A). Furthermore, the DEGs under D, P and DRP stress were mapped onto the pathogen-associated molecular pattern (PAMP) triggered immunity (PTI) pathway (Fig. 3B). We observed the up-regulation of genes encoding calmodulin like (CML24) proteins in all the three cases. *AtHSP90* gene expression was up-regulated in D stress but was down-regulated in P and DRP treated plants (Fig. 3B). Additionally, *AtRbohD* and *AtMPK3* expression was enhanced under both P and DRP treated plants though the expression was higher in the latter case (Fig. 3B). *AtRbohD* gene product has been implicated in ROS production in PAMP-induced defense and is known to act downstream of MAP kinases (MPK3) to positively regulate callose deposition^34^. On the other hand, pathogens use effectors to suppress plant immunity^35–38^. HopN1 effector targeted degradation of plant *PsbQ* gene (involved in photosynthesis) is a contributory factor for Pst DC3000 virulence in susceptible plants^39^. Our study showed an up-regulated expression of *PsbQ* in DRP plants (Fig. 3C) and thus reflected activation of a possible mechanism by which DRP plants can evade effectors and sustain downstream defenses. Besides, some pathogen virulence factors also target plant genes and assist in pathogen entry and virulence into the plant cell. For example, coronatine released by Pst DC3000 suppresses the expression of *AtMyb51* ^40^. DRP stressed plants exhibited higher expression of *AtMyb51* (Fig. 3C) which implicates an upsurge in the glucosinolates and callose deposition as a part of the basal defense in infected plants. AtMyb51 enhances the expression of cytochrome P450 monooxygenases involved in glucosinolate metabolism^41^ and also mediates callose deposition^42^.

We also noted four common genes with a tailored expression between P and DRP treated plants (Supplementary Fig. S7B). As stated in previous section, these genes encoding beta amylase 5 (*AtBAM5*), WRKY DNA binding protein 54 (*AtWRKY54*), a hypothetical protein and short chain dehydrogenase reductase 3a (*ATSDR3*) were up-regulated under P only stress while these were down-regulated under DRP stress (Supplementary Fig. S7B, Supplementary File S2). The differential expression of these genes is in concurrence with the literature^43^ and this activated defense is likely one of the many reasons for reduced pathogen number in DRP (over P only stressed plants).

Since the previous results indicate that plants respond to drought stress and recovery in a distinct way and the pathogen infection at this stage altered the balance of global transcriptome, we proposed that even the metabolism of some prominent metabolites could be altered. Based on previously published literature and the pathway analysis of the transcriptome obtained in our study, we propose that three pathways viz., proline and polyamine metabolism and sugar transport could be altered in DRP over P stressed plants. Proline level is reported to increase during D and P stress^44, 45^. We observed up-regulation of *AtP5CS1* (involved in proline biosynthesis) and down-regulation of *AtProDH2* (involved in proline breakdown) under D stress, which was consistent with the previous study (Supplementary Fig. S13A). During P stress, we observed a down-regulation of *AtP5CS1* but an up-regulation in *AtPRODH2* and *AtSRO5* (which represses expression of P5C dehydrogenase) expression (Supplementary Fig. S13A). These results are in line with earlier reports^46, 47^. Upon re-watering, proline has been reported to revert to non-stress levels (Supplementary Fig. 2E)^7, 9, 13^. In the current study, DRP plants exhibited up-regulation of *AtProDH1* and *AtSRO5* indicating higher catabolism of proline and also implies augmentation of P5C in mitochondria (Supplementary Figs S2F, S13A, S14, S15). Increased P5C synthesis in mitochondria by induction of proline dehydrogenase (*AtProDH1* and *AtProDH2*) and delta-ornithine aminotransferase (*AtδOAT*, involved in the synthesis of P5C from ornithine) encoding genes, had been implicated in defense response of *Nicotiana benthamiana* and *A*. *thaliana* against bacterial pathogens^23^. *AtProDH1* gene was previously shown to be induced under rehydration and also in response to exogenous proline^29^.

We also observed an up-regulated expression of *AtP5CDH* in DRP plants (it was down-regulated in P stressed plants) which implicates higher production of glutamate semialdehyde (GSA). GSA is transported out of mitochondria and in exchange proline is imported from cytosol through the action of unknown glutamate/proline antiporters^48^. Consistent with this, other studies also indicated that after rehydration, accumulated proline is transported to mitochondria and is catabolized due to up-regulation of *AtProDH1* and *AtP5CDH* -mediated step^6, 8^.

**Figure 4:**
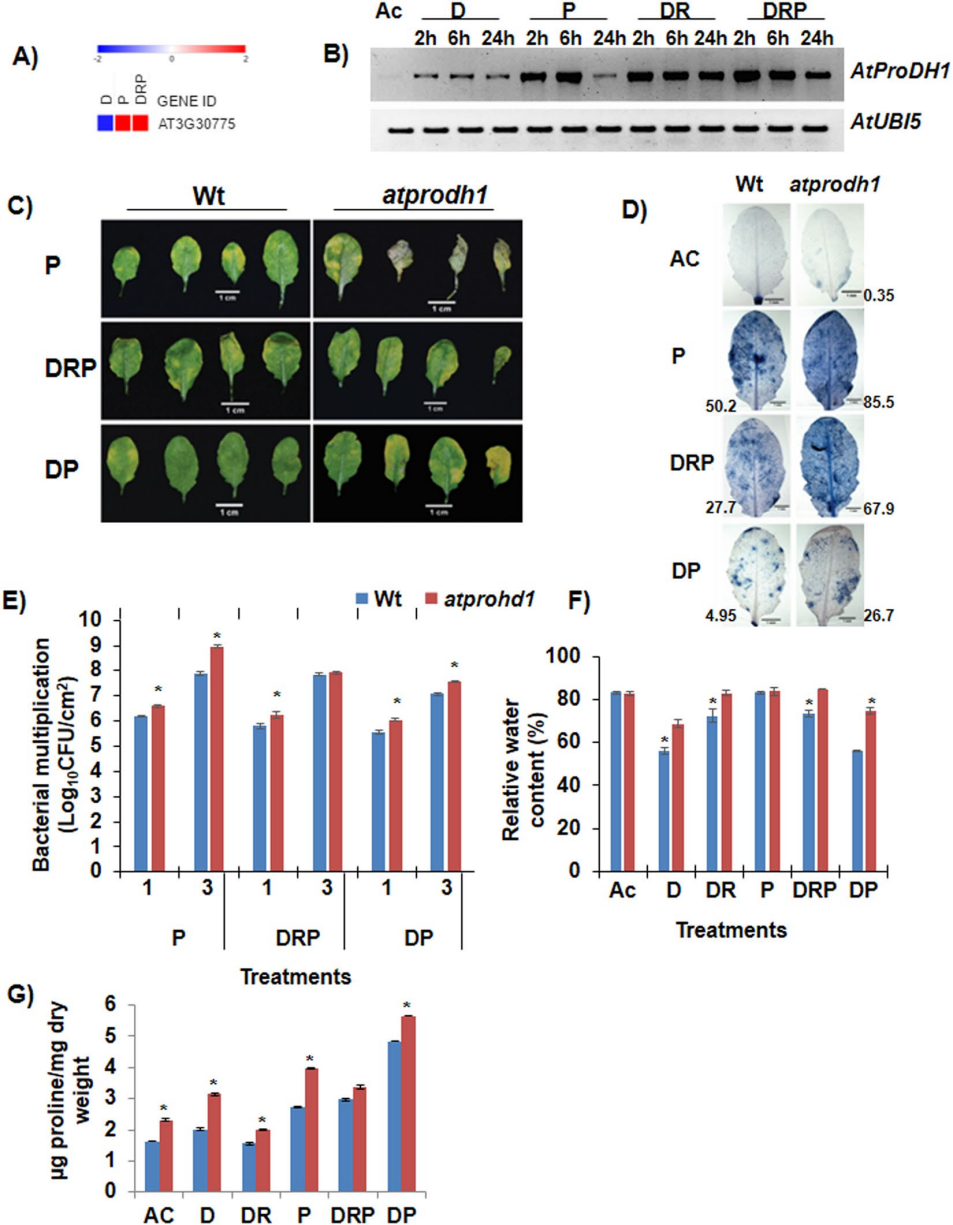
*AtProDH1* gene modulates plant tolerance under combined stress. *atprodh1* mutant and wild-type (Wt) plants were exposed to individual drought (D, at 40% FC), pathogen (P, at 10^4^ CFU/mL) and drought recovery (DR) and combined drought recovery-pathogen (DRP) and drought-pathogen (DP) treatments. Heat map showing expression pattern of *AtProDH1* gene (AT3G30775) was drawn using linear fold change values (D over C and P, DRP over M) from microarray-based transcriptome profile and is presented here (**A**). Colour bar in blue and red shows down- and up-regulated expression respectively. *AtProDH1* transcript expression is shown in WT plants under D, P, DR, DRP and DP stress at 2, 6 and 24 hpt. Semi-quantitative reverse transcriptase PCR was performed with 50 ng of total RNA for 34 cycles. Ubiquitin (*AtUbi5*) gene expression was studied as a reference in the respective samples and is presented as loading control (**B**). Diseased leaves exhibit chlorosis or cell death. Disease phenotype was captured at 4 days post treatment (dpt) in WT and *atprodh1* mutant plants under individual and combined stress treatments and is presented for four biological replicates (**C**). Disease-associated cell death was captured using trypan blue staining at 3 dpt in Wt and *atprodh1* plants in three biological replicates. The extent of cell death is directly proportional to the intensity of blue colour and is represented as fold change over absolute control along each image (ImageJ software) (**D**). Bacterial multiplication in inoculated plants (P, DRP and DP) was monitored at 1 and 3 dpt. Bars represent the mean and SEM for eight biological replicates. Statistical significance for a particular treatment in the mutant plant was determined over respective Wt using student’s *t*-test. Asterisk represents statistically significant value at P < 0.05 (**E**). Relative water content (RWC) in Wt and mutant plants under different treatments was assessed at 24 hpt. Data represent the mean and SEM for three biological replicates. Asterisk represents statistically significant value at P < 0.05 based on Student’s *t*-test between well-watered plants and other treatments (**F**). Proline content in Wt and *atprodh1* plants under different treatments was assessed at 24 hpt. Data are the mean of two biological replicates from one experiment. Bars represent mean ± SEM. Student’s *t*-test was applied to calculate statistical significance in mutant plants over respective Wt treatment. Asterisk represents statistically significant value at P < 0.05 (**G**). Wt, wild-type; AC, absolute control; D, drought; P, pathogen; DR, drought-recovery; DRP, drought-recovery-pathogen; DP, drought-pathogen; 1 and 3, 1 and 3 dpt.

In this regard, *ProDH1* is a likely candidate contributing to ‘shared response’ of plants undergoing combined stress. Its tailored expression indicates a differential role in combined and individual stresses (Fig. 4A) despite its function known under D or P stress, its role under combined DRP has not yet been characterized. We further functionally characterized the role of *ProDH1* (Salk 119334 C, Supplementary Fig. S16) during combined DRP stress. During Pst DC3000 infection, *atprodh1* mutant plants were more susceptible and showed enhanced disease symptoms as compared to the control plants, and this data corresponds to the earlier report^49^. Importantly, the quantitative analysis of bacterial multiplication showed more growth of Pst DC3000 which was accompanied by a more drastic cell death in the leaves of DRP stressed *atprodh1* mutant when compared to the wild-type plants (Fig. 4). *ProDH1* role was also evident under DP combined stress, where its absence imparted susceptibility (Fig. 4). This indicates a weakened defense in DRP stressed *atprodh1* mutant plants. The susceptible phenotype of *atprodh1* plants is known to be associated with the higher content of endogenous proline in these plants under P, DRP and DP stresses (Fig. 4F). *atprodh2* mutants, on the contrary, showed enhanced tolerance to individual and combined stresses (Supplementary Figs S16 and S17). Thus, the common response of *ProDH1* expression during DR, P and DRP stresses holds the key in regulating plant defenses during different combined stresses. Taken together, we propose a model wherein the signaling of proline degradation and transport during DR is one of the major contributors to P5C build-up in DRP plants (Supplementary Fig. S15). P5C production is known to be involved in imparting broad-spectrum defense responses.

Furthermore, the Arabidopsis accessions for polyamine biosynthesis and catabolism related genes were retrieved from literature and DEGs under D and P stress and DRP treatment were mapped onto the polyamine pathway. Notably, our results revealed an up-regulation of genes involved in polyamine biosynthesis in individual D (spermidine synthase, SPDS2) and P (arginine decarboxylase, ADC2) stresses (Supplementary Fig. S13B). However, DRP plants exhibited an up-regulation of the catabolic gene, polyamine oxidase (*AtPAO1*) and down-regulation of spermidine biosynthesis gene, *AtSPDS2* (Supplementary Fig. S13B). AtPAO1 catabolizes SPM to produce SPD^50^. In accordance with our results, the speculated increase in Spd and Spm levels could be associated with recovery from water stress^51^. It thus appears that the spermidine production in a catabolic reaction is more important than its direct synthesis. Furthermore, PAO has been proposed as a major factor for conferring resistance of drought stressed grapevine to *Botrytis cinerea* in a combined stress treatment^52^. Thus, our study provides a clue about the active role of polyamine catabolism in DRP plants.

To probe the involvement of sugar transport in plant defense during recovery, we mapped the DEGs under D, P and DRP stress onto three categories of sugar transport pathways, AtSWEETs, hexose and other sugar transporters (Supplementary Fig. S13C). In our study, we observed a consistent up-regulation of *AtSWEET4* under all three treatments viz., D, P and DRP (Supplementary Fig. S13C). Besides, we also observed a down-regulation of *AtSWEET1*, *AtSWEET2* and *AtSWEET12* (Supplementary Fig. S13C). In P only stressed plants, we noted that the genes encoding for *AtSWEET1* and *AtSWEET13* were up-regulated and the genes encoding for hexose transporters major facilitator superfamily protein and nucleotide/sugar transporter family protein were down-regulated (Supplementary Fig. S13C). During DRP stress, *AtSWEET13*, and hexose sugar transporters *AtERD6*, *AtTMT1* and *AtSUC6* exhibited an up-regulated expression pattern (Fig. 3C). The up-regulated expression of *AtSWEET13* could be either a result of recovery^6^ or phytopathogen could influence it^52^. The up-regulation of *AtSWEET13* and sucrose symporter *AtSUC6* suggests that the plants channelize the nutrients during DRP stress and curb availability of nutrients to the pathogen in the apoplast^53, 54^. Our results second with previous literature that during water deprivation, plants repress sugar transport and minimize energy dissipation; however, upon recovery, when the normal growth processes are resumed, the sugar transport from source to sink is activated again^6^. This contributes to the activated defenses in DRP plants.

In summary, our results from transcriptome analysis followed by functional validation using mutants implicate an activation of proline catabolism. Furthermore, the transcriptome analysis showed a relevance of polyamine and sugar transport in DRP plants (Supplementary Fig. S18). These probably define the unique defenses operating in these plants over P or D stressed plants.

### DRP stressed plants showed unique changes in hormone biosynthesis and their signaling over individual stresses

Transcriptome analysis of unique molecular responses under DRP revealed up-regulated expression of genes involved in jasmonate (JA) and ethylene (ET) biosynthesis (Supplementary Fig. S14). ABA signaling related genes were up-regulated, but salicylic acid (SA) signaling related genes were down-regulated in DRP plants (Supplementary Fig. S14). This was consistent with our results on GO categories with ‘response to SA’ being over-represented in down-regulated DEGs in DRP plants (Supplementary Fig. S12). We also observed enrichment of GO ‘SA mediated signaling’ with up-regulated genes during DRP. Our results are further supported by the earlier report on the existence of both SA-dependent and SA-independent defense pathways operating in plants infected with Pst DC3000^55^. This indicates that SA mediates the activated and unique defense responses under combined DRP treatment.

Further, GO analysis for up-regulated genes showed enrichment of ‘ABA responses’ under D stress, SA, JA and ET under P stress (Supplementary Fig. S11). On the other hand, gibberellin and cytokinin responses were enriched in down-regulated genes for D and P stresses, respectively (Supplementary Fig. S12). Down-regulation of growth hormone-related genes and activation of defense related genes thus indicate more robust defense signaling overgrowth processes under D and P stresses. However, in DRP plants, we did not notice such enrichment which again implicates the DRP responses to be very specific.

### Tradeoff between primary metabolism and stress specific pathways in DRP plants

To probe a correlation between primary metabolism and defense pathways, the transcriptomic datasets under individual D, P and combined DRP treatments were associated with gene ontology (GO) biological processes (corrected *p* value threshold as 0.05 using BiNGO). Functional analysis revealed that the transcriptome of all the stresses studied was enriched with GO category ‘response to stress’ implying a state of stress realized by plants (Supplementary Figs S11 and S12).

The drought stressed transcriptome was enriched with metabolic process, wherein both up- and down-regulated genes were associated with primary metabolism (Supplementary Figs S11, S12). However, *A*. *thaliana* was demonstrated to exhibit a reduction in growth but more photosynthetic capacity, which resulted in increased carbon surplus under drought stress^56^. In addition, cellular biosynthetic process, RNA and ribosomal metabolic processes and ABA-mediated signaling were up-regulated while GA-mediated signaling pathways were repressed during D stress. Such scenarios implicate an activated defense and reduced growth-related processes under D stress.

On the other hand, GO analysis of up-regulated genes under individual P stress displayed an enrichment of primary metabolic and signal transduction processes (Supplementary Fig. S11). It has been previously suggested that increased primary metabolism involving synthesis or degradation products of carbohydrates, amino acids and lipids, possibly moderates signal transduction cascades that lead to plant defense responses^57^. Up-regulated genes from P transcriptome data also correspond to enriched SA and JA-mediated signaling processes. GO analysis of down-regulated genes, showed an enrichment of the photosynthesis, chloroplast organization and chlorophyll biosynthesis which is in accordance to pathogen-mediated chlorosis and interruption of photosynthesis.

Taken together, we infer that plants strike a balance between the cell maintenance and defense pathways while responding to DRP. Under these circumstances, the activation of defense-related pathways is counter-balanced by the repression of genes involved in growth-related primary metabolic pathways^56, 58–60^. Evidences show that mutants with constitutive defense exhibit suppression of growth-related pathways and reduced growth^61–63^. During DRP stress, repression of stress associated processes including protein folding and refolding and SA-mediated processes were also observed (Supplementary Fig. S12). However, under DRP stress, the up-regulated genes were enriched with processes namely cell communication, organic acid transport and SA-mediated signaling (Supplementary Fig. S11).

## Conclusions

In the present study, we analyzed the transcriptome profile of D, P and DRP treated plants. We compared the transcriptional changes in D and DRP plants with DR (from the previous study) plants and observed that the majority of the drought-induced transcriptomic changes were restored in DR and DRP. We also noted the persistence of some drought influenced transcriptomic changes through recovery. Overall, the transcriptional dynamism was maintained along gradients of host water status during pathogen infection. We attribute that the specific transcriptome changes in DRP, when compared to the pathogen, could be one of the reasons for the reduction in pathogen multiplication. Altogether, the results show that DRP plants underwent a drastic transition state different from that of control, drought and pathogen infection. Polyamine catabolism and the role of SA-mediated signaling were found to be distinct in DRP. Proline catabolism, on the other hand, forms a part of common plant responses elicited during individual and combined stresses. Further, we conclude that DRP plants maintain a balance between primary metabolism and defense processes.

## Electronic supplementary material

Supplementary Figures

Supplementary File S1

Supplementary File S2

Supplementary File S3

Supplementary File S4
