# Supplementary material for: Transcriptome changes in Arabidopsis thaliana infected with Pseudomonas syringae during drought recovery
Source: Sci Rep. 2017 Aug 22;7:9124. doi: 10.1038/s41598-017-09135-y (PMC5567376; doi:10.1038/s41598-017-09135-y)
Supplement: Supplementary file 1 — Supplementary Figures [file 41598_2017_9135_MOESM1_ESM.pdf]

Supplementary Figures 1 to 18

Transcriptome changes in *Arabidopsis thaliana* infected with *Pseudomonas syringae* during drought recovery

Aarti Gupta and Muthappa Senthil-Kumar*

National Institute of Plant Genome Research, Aruna Asaf Ali Marg, New Delhi, India

Supplementary Figure 1

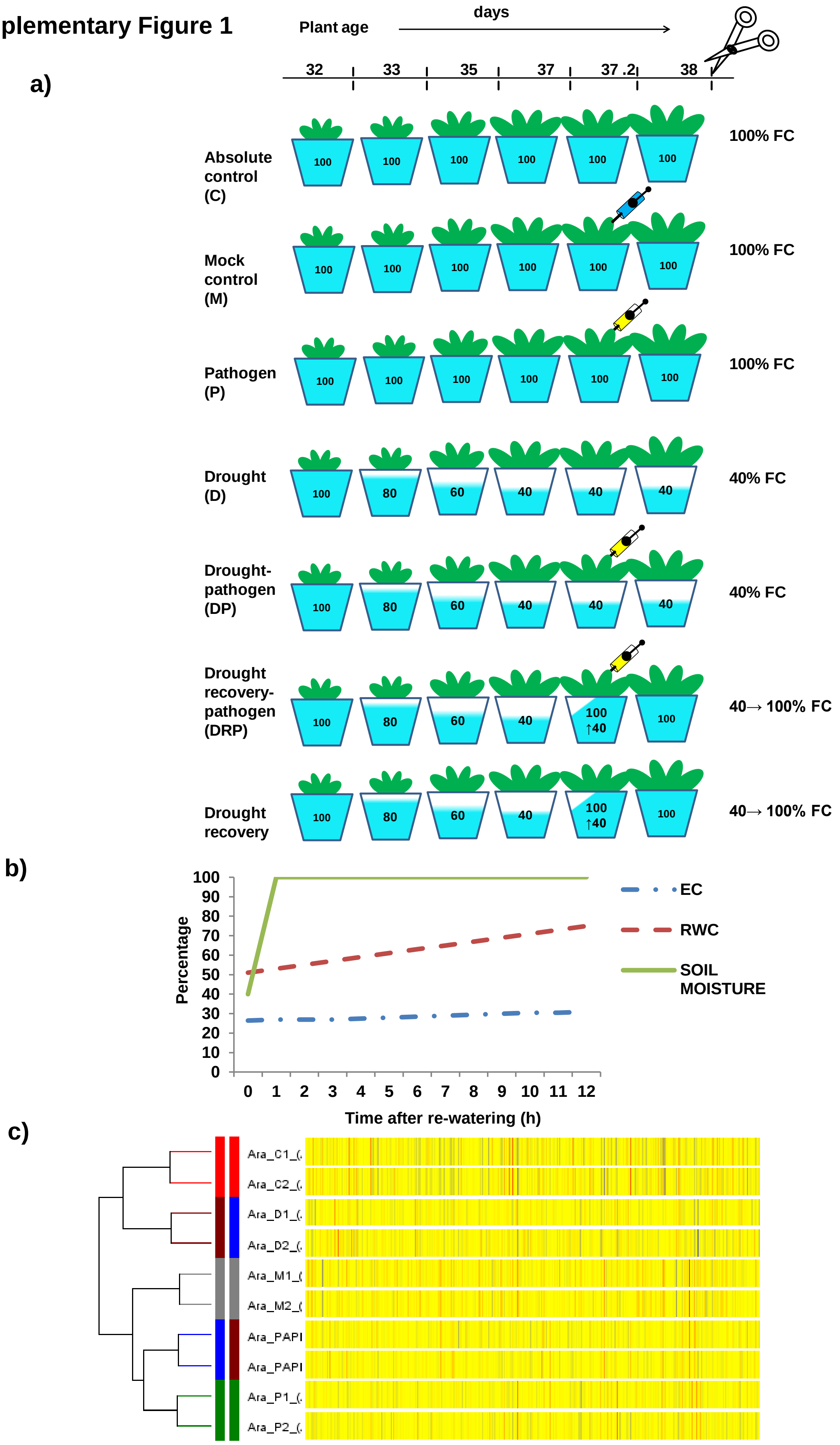

Supplementary Figure 1: Experimental layout for drought-recovery-pathogen treatment in *A. thaliana*. Pictorial representation shows methodology adopted for drought-recovery-pathogen treatment and individual drought and pathogen stress treatments. *A. thaliana* (32-d post germination) was subjected to drought stress (soil moisture content at 40% field capacity, FC; equal to Ψw -3.9 MPa), measured and maintained by gravimetric method. Once potted plants accede to 40% FC, they were bottom-watered for 2 h. Two hours after start of re-watering, Pst DC3000 was syringe infiltrated at the concentration of 5 X 10^3 CFU/mL for transcriptome analysis and 1 X 10^4 CFU/mL for physiological assays. Leaf samples, at the same developmental stage were harvested from 38-d-old plants (24 hpt) and observations were recorded. Soil moisture level is depicted as the height of blue color in pot, the upward blue syringe shows mock inoculation with water, yellow syringe indicates inoculation with pathogen, scissors mark the time of sample harvest, values marked over each pot correspond to the FC of that pot **(A)**. We also exposed a batch of drought stressed plants maintained under similar conditions, to drought recovery. The DR treated plants were used for the qRT-PCR based validation of the genes. Graphical simulation of expected alteration in stress parameters over 12 h period after re-watering is presented here **(B)**. In order to show the relatedness of replicates and different treatments, hierarchal clustering based on Euclidean distance was performed using signal intensity values from array probes (hybridization) in different conditions including absolute control (C1, C2), mock (M1, M2), drought (D1, D2), pathogen (P1, P2), combined drought-recovery-pathogen treatment (PAPR1, PAPR2) and is represented here **(C)**. Olivas et al. (New Phytol 2016, 1344-1356) and Coolen et al. (Plant J 2016, 86, 249–267) have referred pathogen infection after 1d drought recovery as sequential stress. However, in our study pathogen was inoculated at the time of recovery and this may be referred as combined treatment. Cartoons are not to scale.

The Drought recovery-pathogen treatment was also used to demonstrate *Rice-Magnaporthe* intraction by Bidzinski et al. (Front Plant Sci 2016, 7:1558)

Supplementary Figure 2

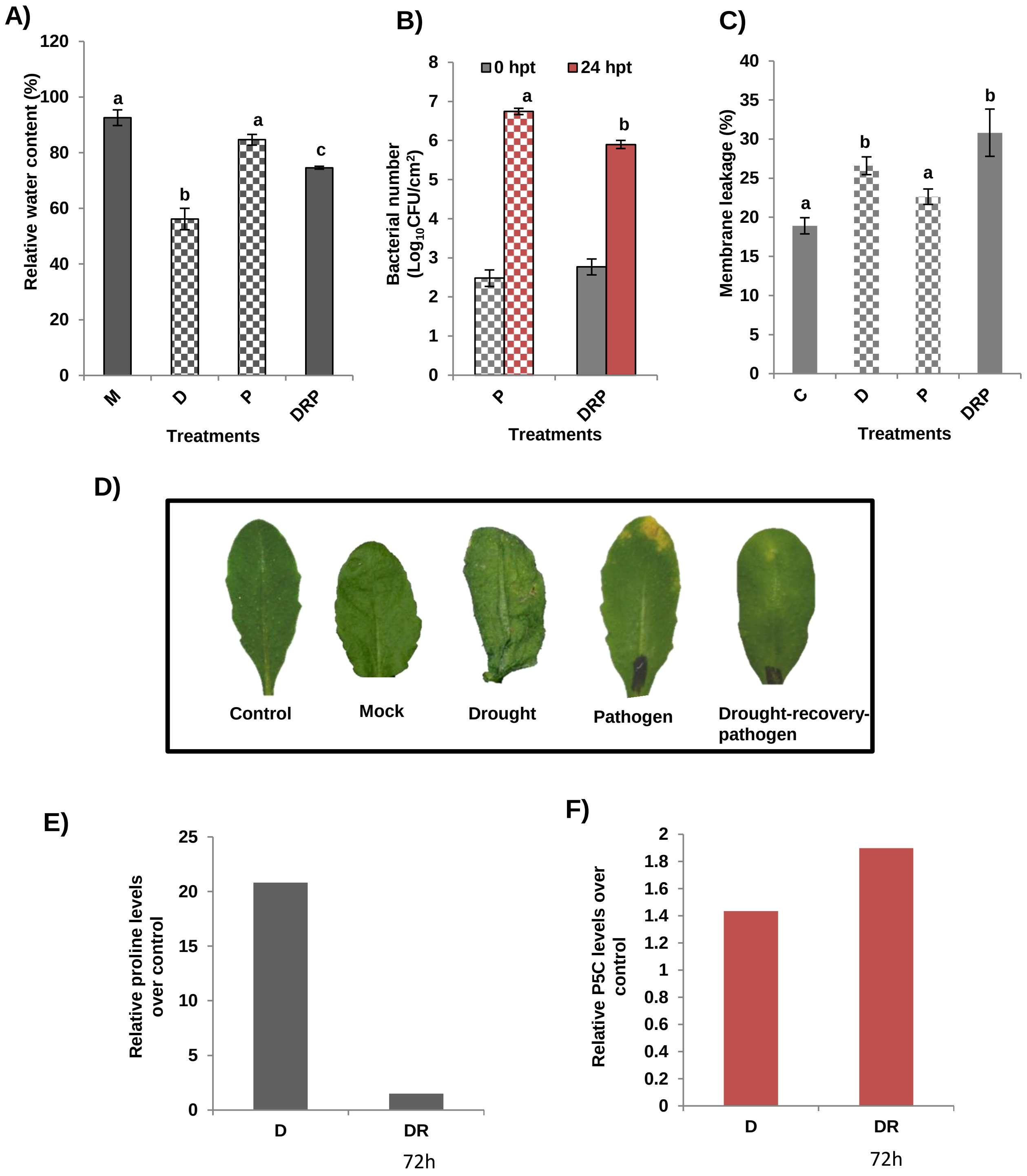

Supplementary Figure 2: Impact of DRP treatment on *A. thaliana*. Plants were exposed to individual and combined stress treatments as depicted in Supplementary Fig. S1. For physiological assays, the pathogen was infiltrated at the concentration of 1 X 10^4 CFU/mL. In order to study stress interaction of D or DR on pathogen, bacterial multiplication numbers were quantified. Drought levels and pathogen influence on plant water relations during interaction were measured by leaf relative water content (RWC). Net impact of stress interaction on plants was assessed by membrane leakage. Leaf samples were harvested at 24 hpt and stress impact was established with aforementioned phenotypic and physiological evaluations. RWC is shown under different stress conditions **(A)**. *In planta* bacterial multiplication numbers are presented from P only and DRP stressed plants **(B)**. Membrane leakage (%) in leaves is shown under different stress conditions **(C)**. Phenotype shows wilting and/or disease symptoms in stressed plants and was recorded at 24 hpt **(D)**. Individual stressed plants should be compared against absolute or mock control and DRP treated plants should be compared with respective individual stresses. Data presented is the average from 6 biological replicates with error bars representing ± SEM. Significance was calculated using one-way ANOVA. Different letters denote significance at p Different letters denote significance at $p<0.05$ (Tukey's multiple comparison test). Data represented in the checked bar graphs is adopted from previous study (Gupta et al., Front Plant Sci 2016, 7:686 doi: 10.3389/fpls.2016.00686).

Ratio of proline and Δ^1-Pyrroline-5-Carboxylate (P5C) levels under drought and drought recovery (DR) are presented over control. Data was retrieved and reprocessed from Miller et al (J Biol Chem 2009, 284:26482–26492) **(E, F)**.

AC, control plants maintained at 100% FC; M, plants maintained at 100% FC infiltrated with water; D, drought stressed plants maintained at 40% FC till the end of the experiment; P, plants maintained at 100% FC infiltrated with pathogen; DRP, recovery from drought stress (40% FC to 100%) followed by pathogen infiltration and were maintained at 100% FC till the end of the experiment.

Supplementary Figure 3

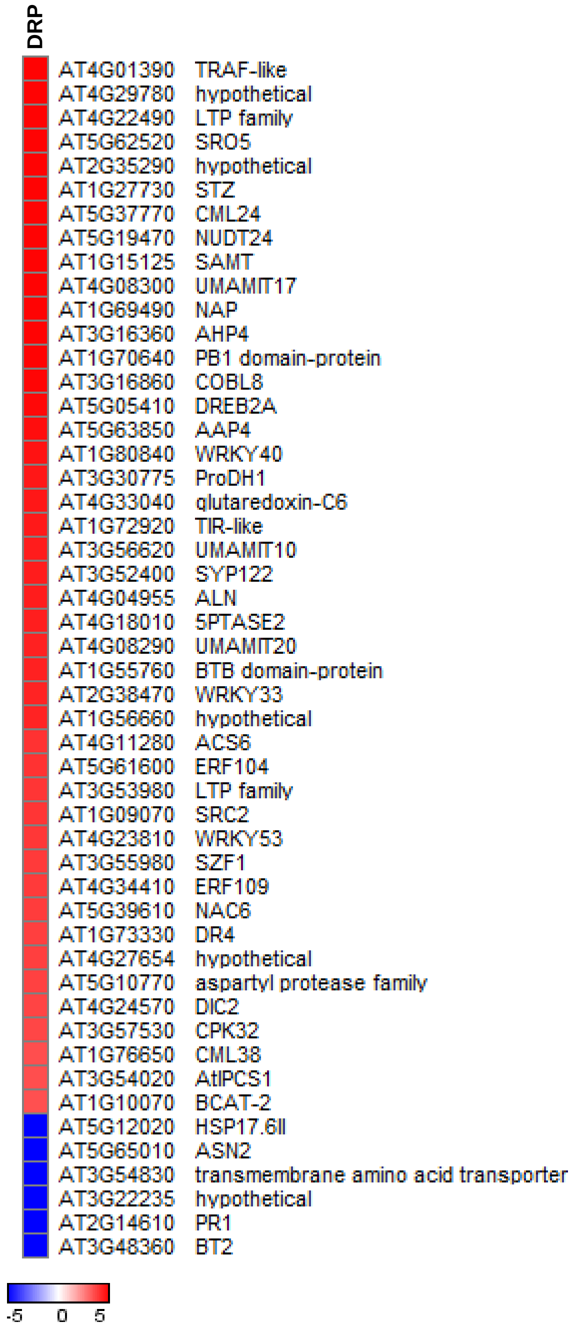

Supplementary Figure 3: Expression profile of highly regulated transcripts under DRP stress. Fold change values for 50 top-most differentially expressed transcripts under DRP stress were computed against mock control. Fold change values were used to plot heat map. Color scale shows up-regulated genes in red and down-regulated genes in blue. Corresponding details for the gene IDs presented in the figure are provided in Supplementary File S2.

Supplementary Figure 4

A)

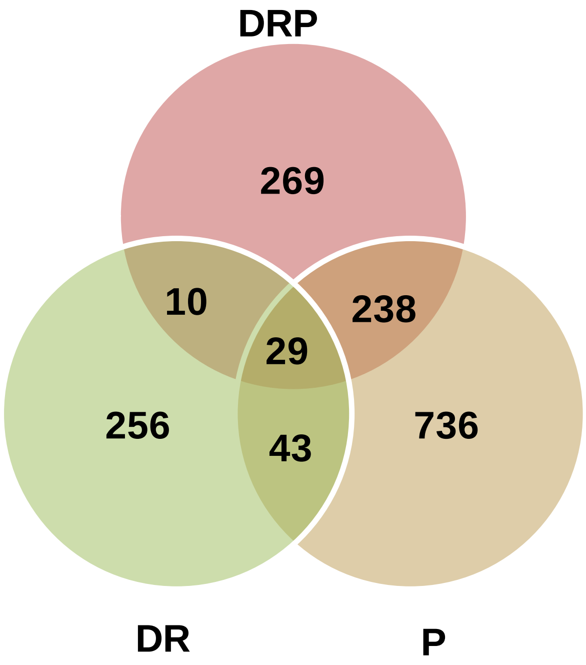

B)

Number of genes	DR	P	DRP
13			
1			
3			
12			

C)

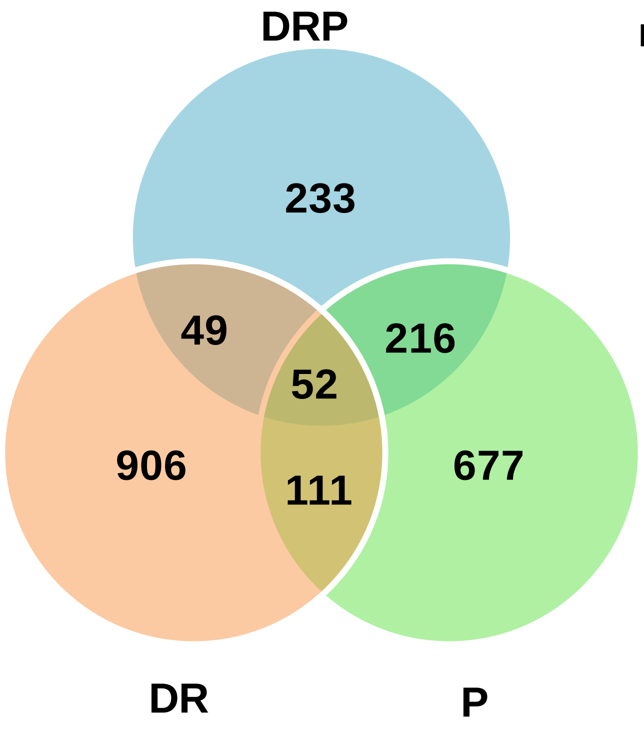

D)

Number of genes	DR	P	DRP
12			
4			
9			
25			
2			

Supplementary Figure 4: Summary of transcriptome profile of *A. thaliana* exposed to drought-recovery-pathogen treatment relative to individual stresses. Differentially expressed genes (DEGs) under P and DRP stress treatments were identified from present study as depicted in Figure 1 and Supplementary Figures 1, 2. Differentially expressed genes under drought recovery (DR) were acquired from literature. Venn diagram between DEGs in combined DRP stress, individual P stress and drought recovery (DR) adopted from Oono et al (Plant J 2003 34, 868–887) **(A, B)** and Coolen et al (Plant J 2016 86, 249–267) **(C, D)** revealed presence of transcripts exclusively under DRP stress and were regarded as ‘unique’ genes and ‘common’ transcripts shared between individual and combined stresses. Expression profile of genes common among DR, P and DRP is presented in the form of color blocks. Numbers corresponding to each color block represent number of genes in respective category. Green color represents up-regulation and red corresponds to down-regulation.

Supplementary Figure 5

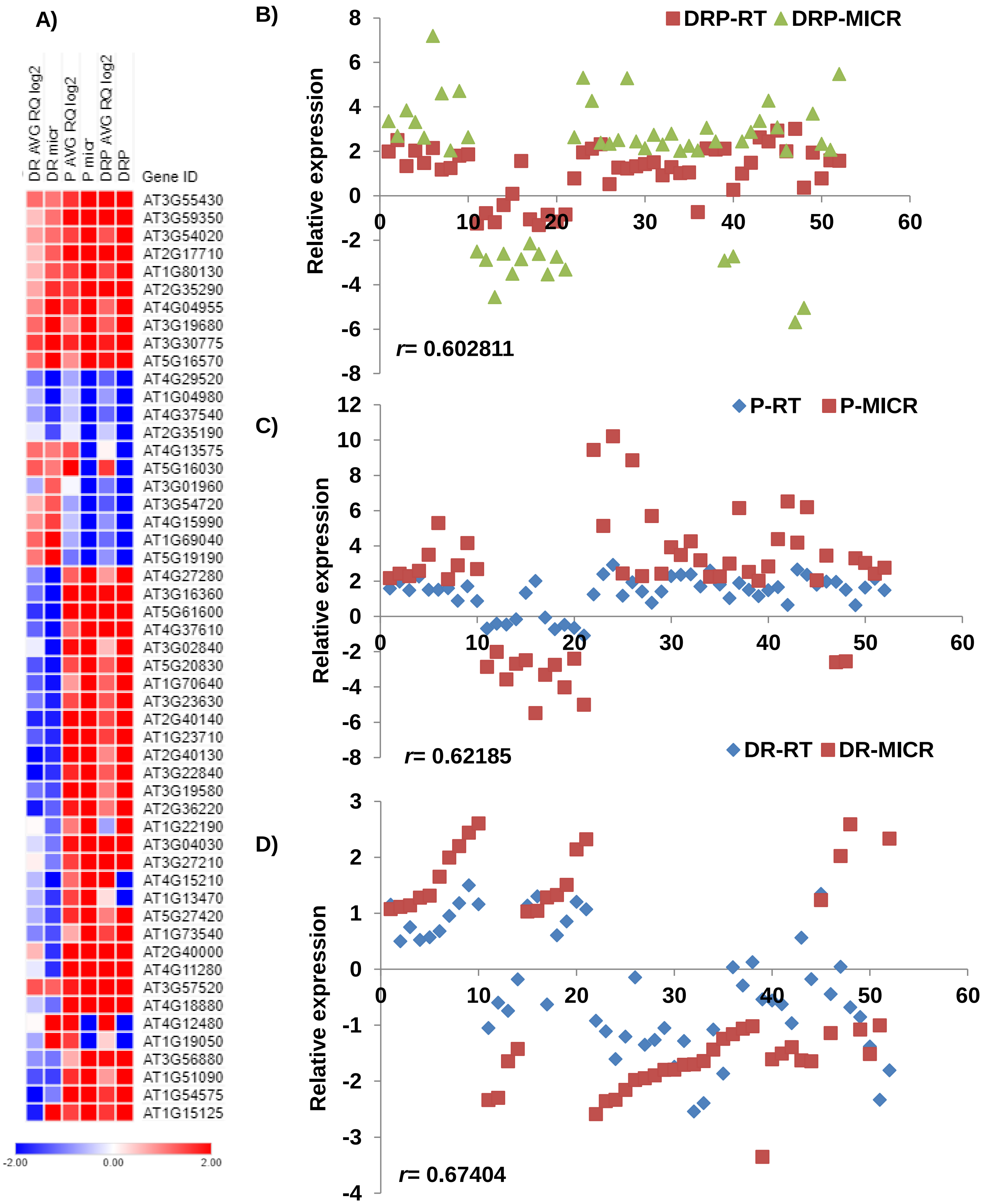

Supplementary Figure 5: RT-qPCR validation of common genes transcriptomic data from DRP, P or DR treated plants. Differentially expressed genes (DEGs) under P and DRP stress treatments were identified from present study as depicted in Figure 1 and Supplementary Figures 1, 2. Through Venn intersection, ‘common’ transcripts shared between individual DR and P and combined DRP treatments were identified and selected to validate the comparison made in Figure 1 by RT-qPCR analysis. For this, *A. thaliana* was exposed to DR, P or DRP stresses and leaf sample was harvested at 24 hpt. Tissue from two plants was pooled to isolate total RNA. Fold change in expression levels relative to the control samples (DR versus absolute control and P and DRP versus mock control) were normalized to *AtACTIN2* gene expression. RT-qPCR based quantification was performed with four biological replicates (and two technical replicates). RT-qPCR based relative quantities of gene expression values was log2 transformed and compared with expression profile of genes obtained through microarray experiment. Comparative expression profile of the selected genes obtained from q-RT-PCR and microarray is presented in the form heatmap (A). Colorbar in blue and red presents down-up regulated genes respectively. Scatter plot represents the comparison of relative fold change values (compared to control or mock treatment) in expression of genes obtained from microarray and RT-qPCR analysis under DRP **(B)**, P **(C)** and DR **(D)** treatments. Represented data are the average of four biological replicates ‘r’ denotes the Pearson correlation coefficient drawn from comparison of the two datasets. Gene names and descriptions for the gene IDs presented in the figure are provided in Supplementary File S4. Details of primers used in the study are provided in Supplementary File S4. Raw data from RT-qPCR experiment is provided in Supplementary File S2. DR; drought recovery, P; pathogen stress, DRP; pathogen stress on drought-recovering plants; MICR, microarray.

Supplementary Figure 6

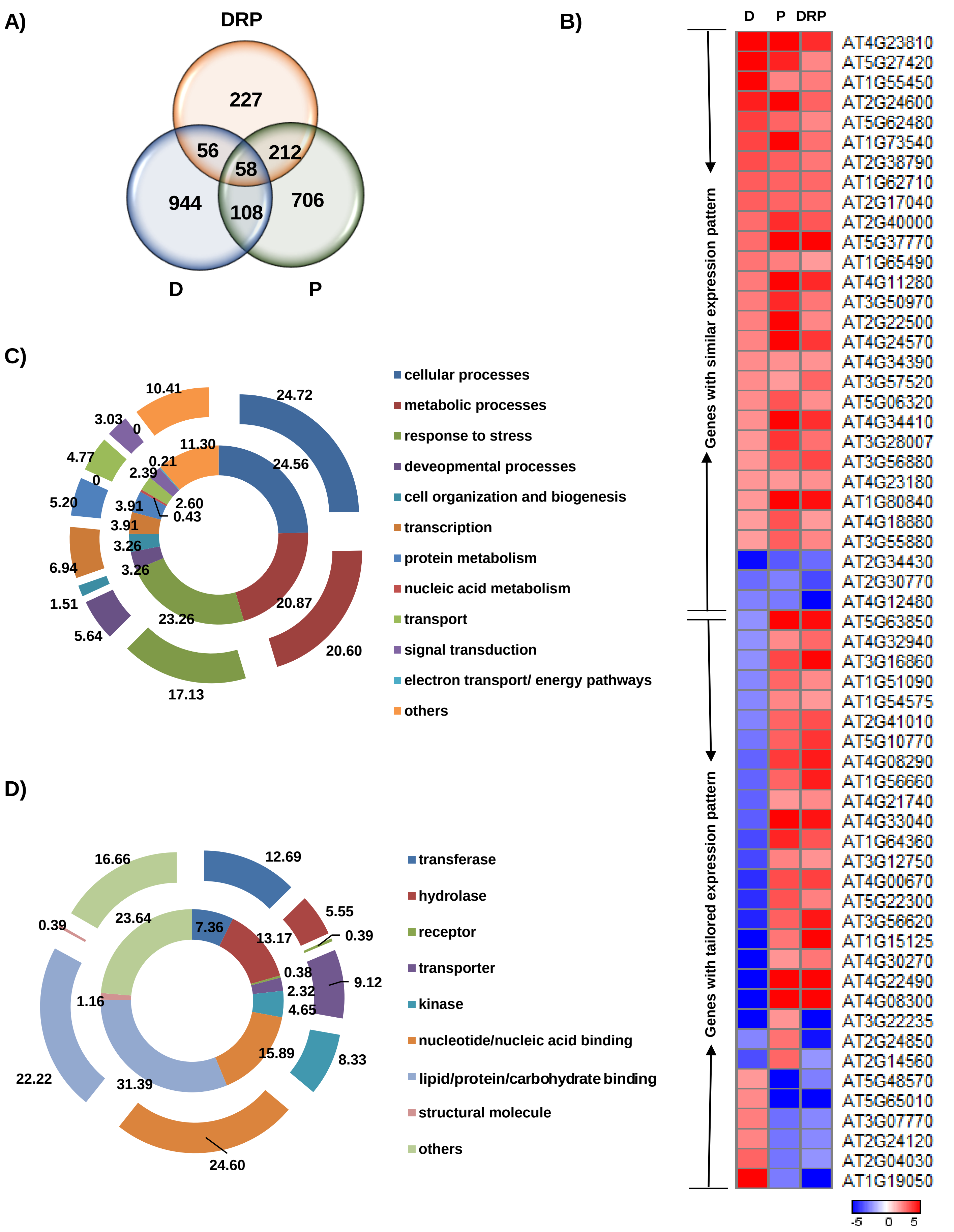

Supplementary Figure 6: Comparative transcriptome profile of *Arabidopsis thaliana* exposed to combined drought-recovery-pathogen and individual drought and pathogen treatments. *A*. *thaliana* was exposed to individual drought (D), *Pseudomonas syringae* pv. tomato DC3000 (Pst DC3000; P) and combined drought-recovery-pathogen (DRP) treatments as outlined in Figure S1. Microarray hybridization on Affymetrix WT gene chip array was conducted using total RNA isolated from leaf samples (38-d-old plants) harvested at 24 hours post treatment (hpt). Differentially expressed genes (DEGs) in each stress treatment were identified in comparison to control conditions and threshold was set at change greater than 2 fold and ANOVA *p* value <0.05. Venn diagram between DEGs in individual and combined stress revealed presence of transcripts exclusively under DRP stress and were regarded as 'unique' genes and 'common' transcripts shared between individual and combined stresses **(A)**. Expression profile of genes common among D, P and DRP is presented in the form of heat map. Common transcripts were categorized as genes with similar expression pattern in all the three stress conditions and as genes with 'tailored' expression pattern under different stresses (up-regulated vs. down-regulated and vice versa) **(B)**. Expression values were used to plot heat maps using GENE-E software (http://www.broadinstitute.org/cancer/software/GENE-E/). Color bar scale shows the fold change range with red and blue color representing up- and down-regulation respectively. Corresponding gene names and descriptions for the gene IDs presented in the figure are provided in Supplementary File S1. Genes unique to the DRP stress were functionally categorized and represented based on gene ontology (GO) biological process **(C)** and GO molecular function **(D)**. Outer disc implies the GO categories for up-regulated genes, inner disc implies GO categories for down-regulated genes.

Supplementary Figure 7

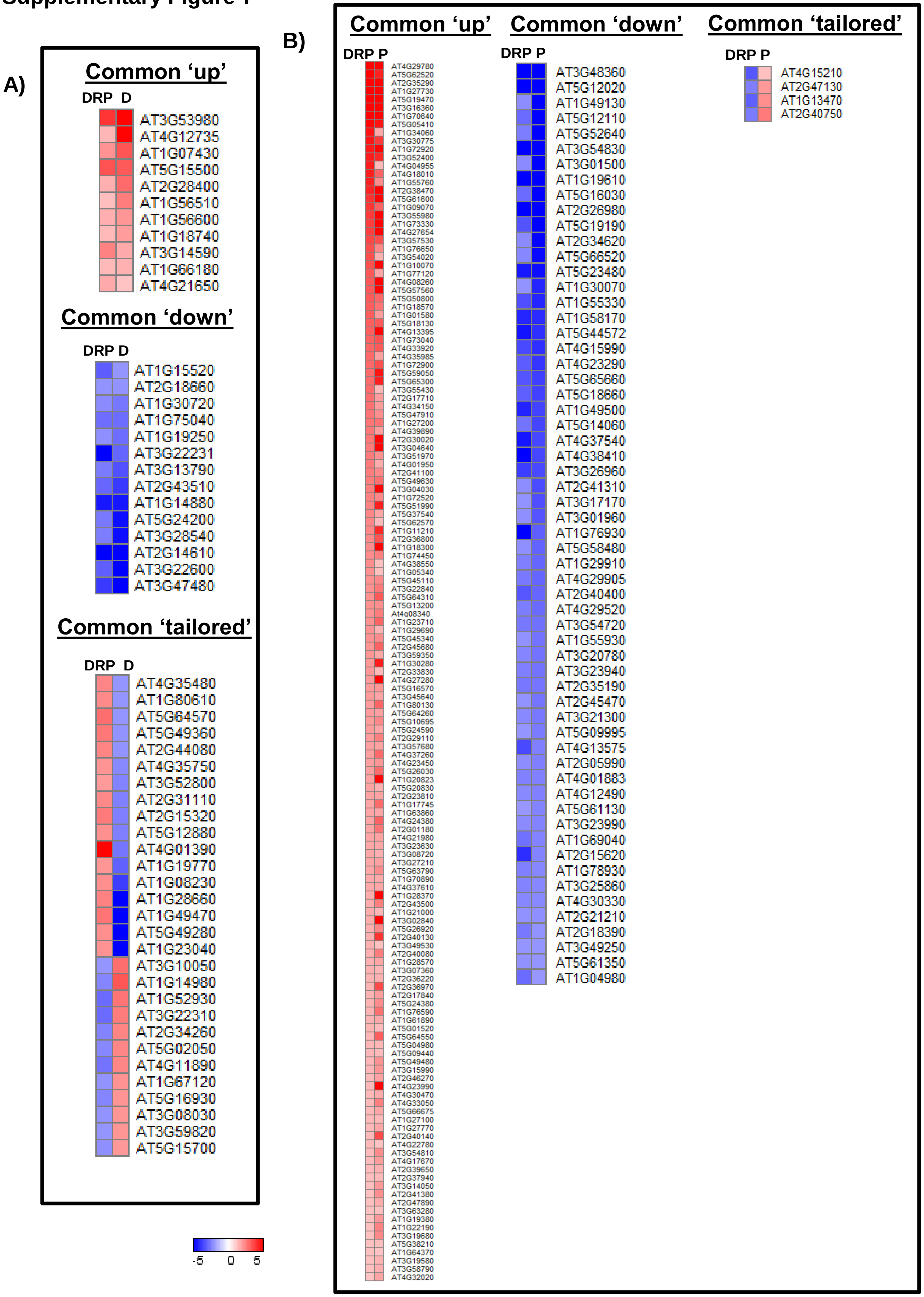

Supplementary Figure 7: Expression profile of common genes between individual and DRP stress. Expression profile of genes common between D and DRP stress **(A)** and P and DRP stress **(B)** is presented in the form of heat map using GENE-E software. Common up, gene common between DRP and D or P stress having up-regulated expression; common down, common genes with down-regulated expression in DRP and D or P stress; common tailored, common genes with up-/ down-regulated expression in DRP and D or P stress. Color scale shows up-regulated genes in red and down-regulated genes in blue. Corresponding details for the gene IDs presented in the figure are provided in Supplementary File S2.

Supplementary Figure 8

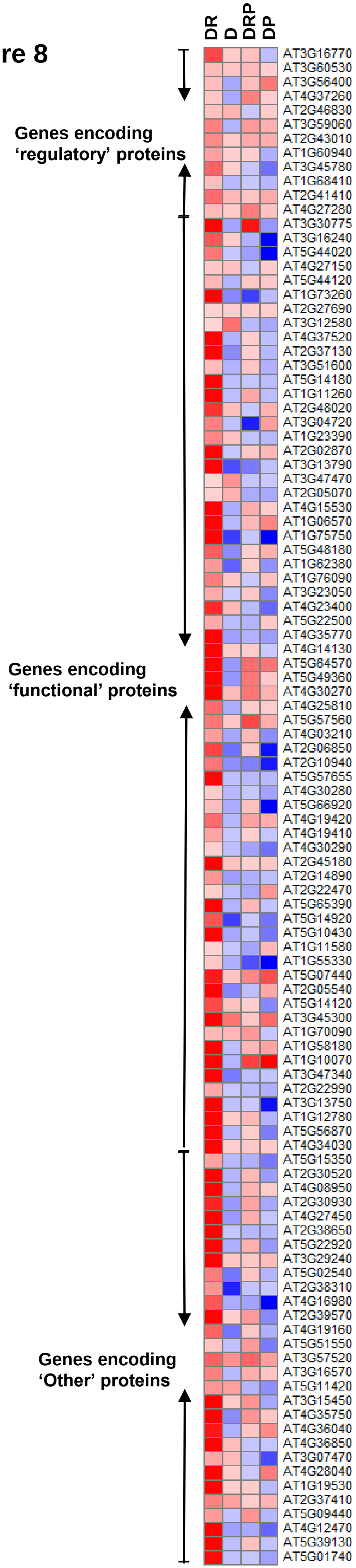

Supplementary Figure 8: Comparative transcriptome profile of DR, DRP, D and DP treatments. Drought recovery related genes were retrieved from the study performed by Oono et al (Plant J 2003 34, 868–887) and the expression pattern was compared with present study whole transcritome data of D, P DRP and DP stressed plants. Heat maps represent expression profile of recovery inducible genes under DR, D, DRP and DP stresses. Differentially expressed genes were compared with recovery-transcriptome (Oono et al Plant J 2003 34, 868–887). Color bar scale shows the fold change range with red and blue color representing up- and down-regulation respectively. Gene details for represented Gene IDs are provided in supplementary file S2.

Supplementary Figure 9

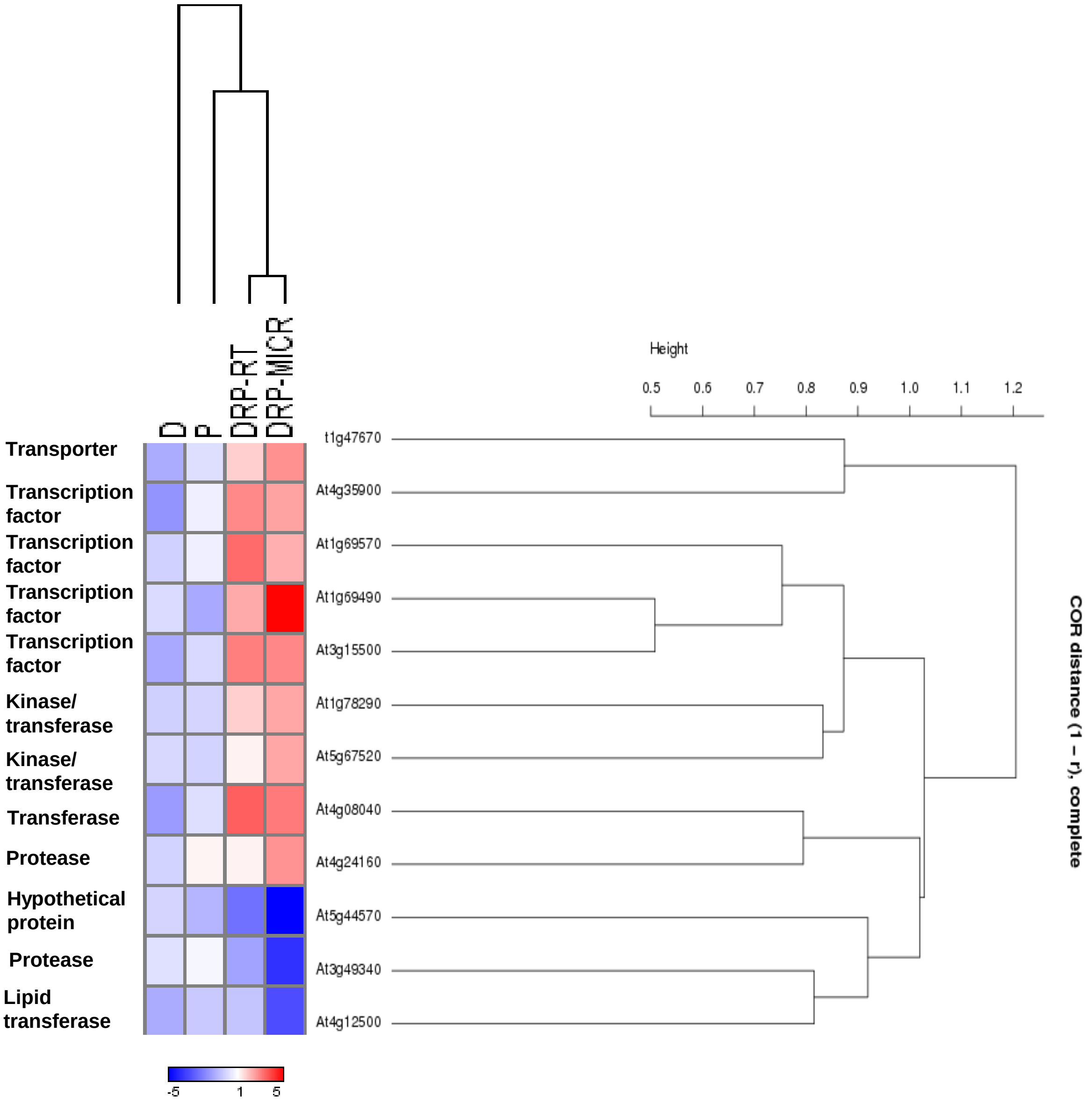

Supplementary Figure 9: RT-qPCR showing validation of unique genes from microarray based DRP combined stress transcriptome profile. Expression profile of highly up- (9 genes) and down-regulated (3 genes) unique genes with probable 'regulatory function' under DRP stress was validated and quantified by RT-qPCR based quantification under individual D and P and DRP stress treatment. Co-expression based gene clustering was done using ATTED-II online tool (http://atted.jp/). Fold change in expression levels relative to the control samples were normalized to *AtACTIN2* gene expression. RT-qPCR analysis was carried out with three biological and two technical replicates. Heat map represents the fold change values in gene expression obtained from RT-qPCR under D, P, and DRP stress treatments. Color bar in red and blue represents up- and down-regulated genes respectively. Dendrogram represents hierarchal clustering between expression values under different conditions based on one minus Pearson correlation. Gene names and descriptions for the gene IDs presented in the figure are provided in Supplementary File S2. Details of primers used in the study are provided in Supplementary Table S1. MICR; expression values from microarray data, RT; expression values from real-time q-PCR experiment.

Supplementary Figure 10

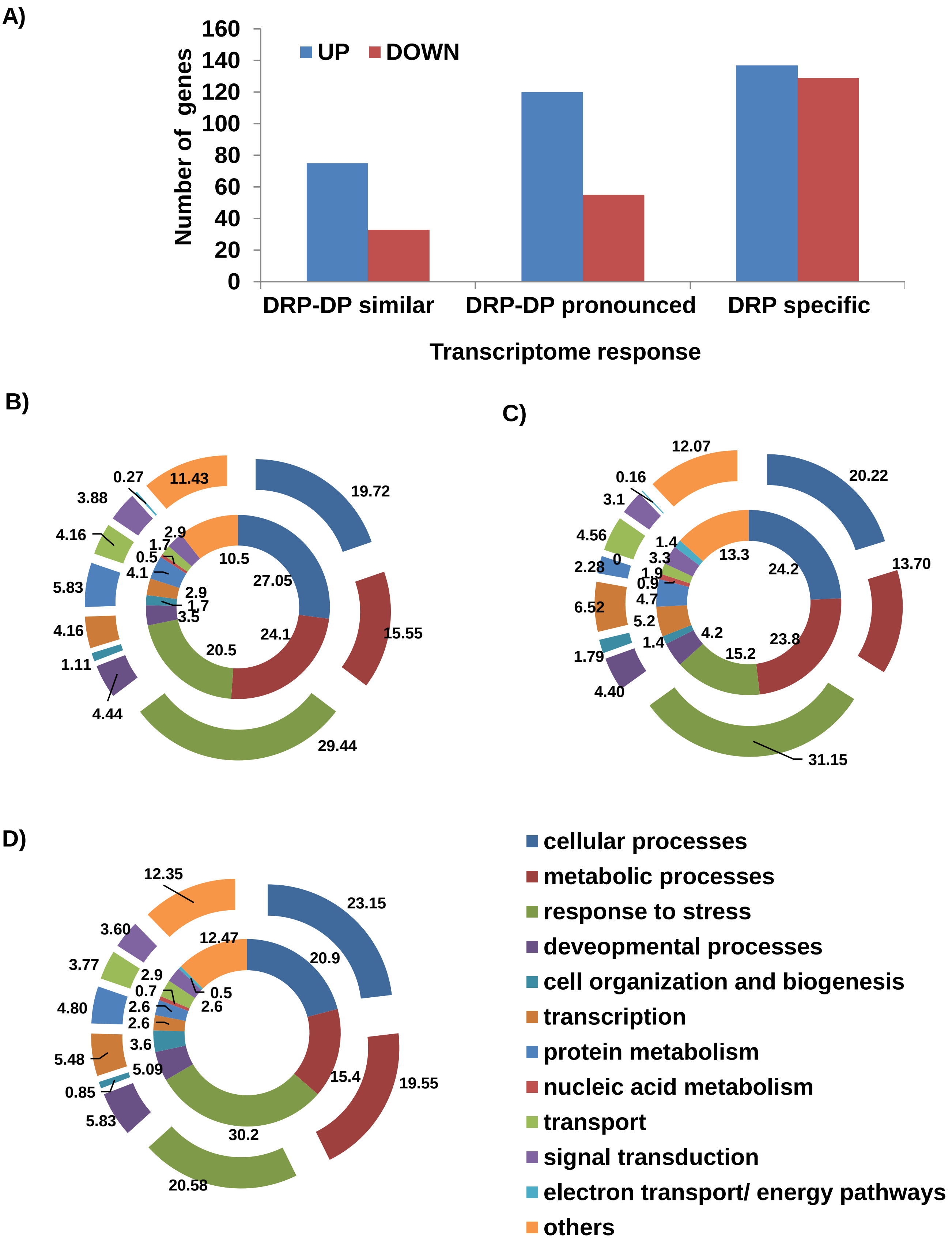

Supplementary Figure 10: Comparative transcriptome profile of *A. thaliana* under combined DRP treatment over DP stress. List of 553 DEGs (2 fold change cut off, p value < 0.05) under DRP stress was compared with genes lists (no fold change cut off, p value < 0.05) from DP combined stress (Gupta et al., Front Plant Sci 2016, 7:686 doi: 10.3389/fpls.2016.00686). Based on the expression profile of genes under two conditions, the genes were grouped as 'DRP-DP similar', when the trend in expression pattern of the gene under DRP treatment was similar to that under DP stress; 'DRP-DP pronounced', when the expression of gene was high under DRP treatment than DP stress and 'DRP specific' when there was no change in expression or opposite (up and/or down regulation) expression under DP compared to DRP treatment **(A)**. These groups were further segregated into up- and down-regulated transcripts. All these groups were classified by GO functional categorization based on biological process, DRP-DP similar **(B)**, DRP-DP pronounced **(C)**, and DRP specific **(D)**. Percent distribution of up- and down-regulated genes across different GO categories are presented in peripheral and inner ring respectively.

Supplementary Figure 11

Gene Ontology Enrichment of up-regulated genes

D **Description**

- primary metabolic process
- biosynthetic process
- organic substance biosynthetic process
- nitrogen compound metabolic process
- macromolecule biosynthetic process
- gene expression
- nucleic acid metabolic process
- ribosome biogenesis
- RNA modification
- ncRNA metabolic process
- rRNA metabolic process
- nucleobase-containing compound metabolic process
- heterocycle metabolic process
- cellular aromatic compound metabolic process
- organic cyclic compound metabolic process
- indole glucosinolate catabolic process
- cellular component biogenesis
- deadenylation-dependent decay
- response to inorganic substance
- response to oxygen-containing compound
- response to water deprivation
- response to osmotic stress
- response to cold
- response to high temperature
- response to alcohol
- response to organonitrogen compound
- response to organic substance
- response to salt stress
- response to abscisic acid
- response to lipid
- response to bacterium
- response to chitin

DRP **Description**

- cell communication
- hormone-mediated signaling pathway
- organic acid transmembrane transport
- response to osmotic stress
- response to wounding
- response to oxidative stress
- response to oxygen-containing compound
- response to organonitrogen compound
- response to organic substance
- response to nitrogen compound
- response to salt stress
- response to chitin
- response to bacterium
- Incompatible interaction
- response to hormone
- response to temperature stimulus
- immune system process
- immune response
- regulation of salicylic acid metabolic process

0 5

P **Description**

- primary metabolic process
- biosynthetic process
- nitrogen compound metabolic process
- aromatic compound biosynthetic process
- organic cyclic compound biosynthetic process
- macromolecule metabolic process
- nucleobase-containing compound metabolic process
- gene expression
- RNA metabolic process
- heterocycle biosynthetic process
- organic substance metabolic process
- oxylipin metabolic process
- oxoacid metabolic process
- transition metal ion homeostasis
- carboxylic acid metabolic process
- organic acid transmembrane transport
- ion transport
- vasculature development
- cell communication
- signaling
- phosphorelay signal transduction system
- hormone-mediated signaling pathway
- jasmonic acid mediated signaling pathway
- salicylic acid mediated signaling pathway
- response to water deprivation
- response to osmotic stress
- response to high temperature
- response to cold
- response to light
- response to UV
- response to hypoxia
- response to ozone
- response to metal ion
- response to alcohol
- response to oxygen-containing compound
- response to reactive oxygen species
- response to oxidative stress
- response to wounding
- response to organic substance
- response to organic cyclic compound
- response to lipid
- response to karrikin
- response to chitin
- defense response
- innate immune response
- response to organonitrogen compound
- response to nitrogen compound
- response to bacterium
- response to molecule of bacterial origin
- incompatible interaction
- response to fungus
- response to hormone
- response to abscisic acid
- response to salicylic acid
- response to jasmonic acid
- response to salt stress
- response to ethylene
- salicylic acid metabolic process
- immune system process
- immune effector process
- plant-type hypersensitive response
- host programmed cell death induced by symbiont
- programmed cell death
- cell death

Supplementary Figure 11: Gene ontology enrichment of up-regulated genes in *A. thaliana* under D, P and DRP stress. Up-regulated DEGs (2 fold change cut off, p value < 0.05) under D, P, stresses and DRP treatment were associated with over-represented GO biological processes. The Biological Networks Gene Ontology (BiNGO; Maere et al., 2005 Bioinformatics 21:3448–3449) was used, with Bonferroni corrected p-value threshold set at 0.05. Based on enrichment values, the respective GO biological processes were represented in the form of heat map for D, P and DRP stressed transcriptome. Color bar scale in white to red represents enrichment score of 0 to 5. List of all enriched GO biological processes corresponding to up-regulated genes in each stress category is presented in Supplementary File S3.

Supplementary Figure 12

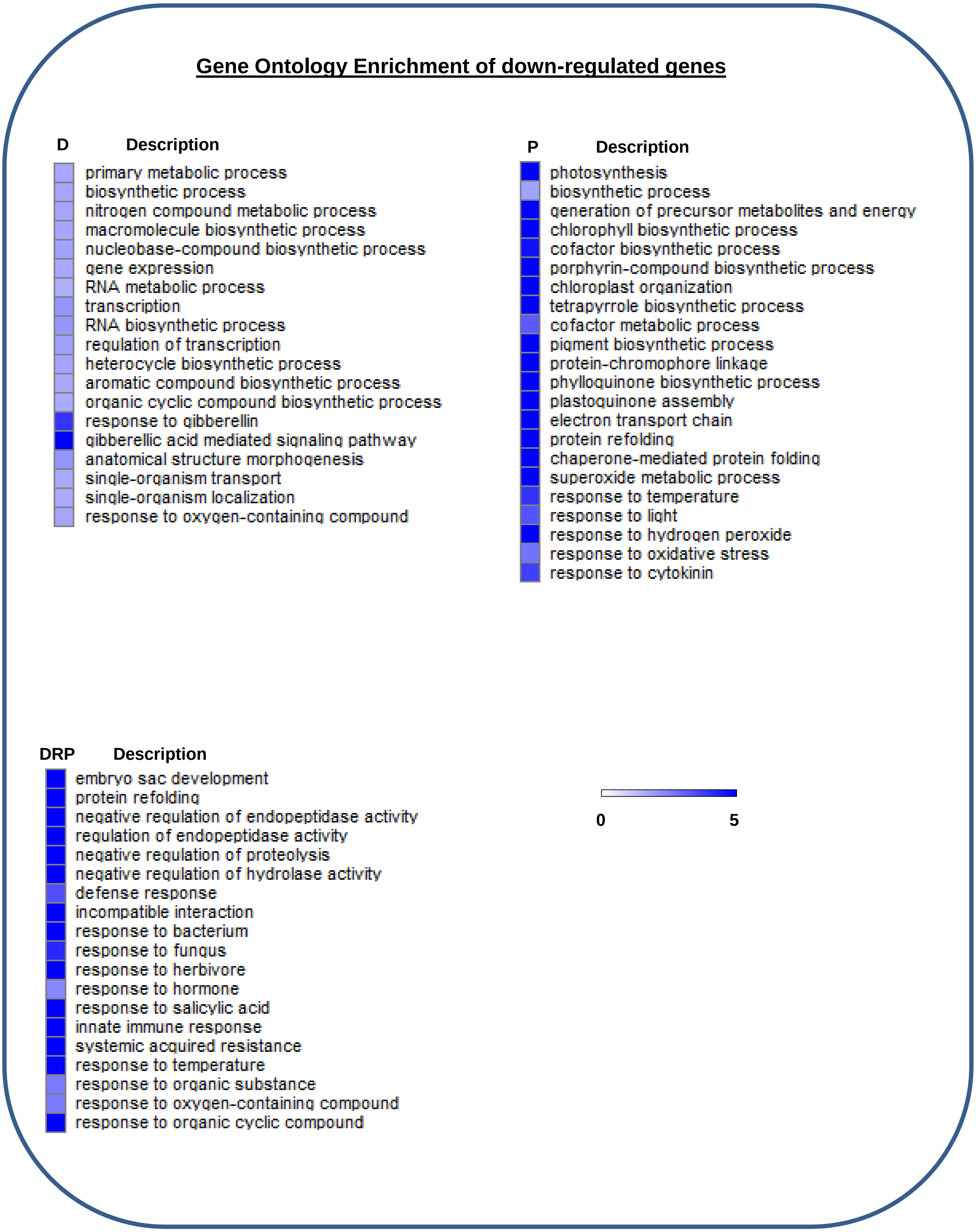

Supplementary Figure 12: Gene ontology enrichment of down-regulated genes in *A. thaliana* under D, P and DRP stress. Down-regulated DEGs (2 fold change cut off, p value < 0.05) under D, P, stresses and DRP treatment were associated with over-represented GO biological processes. The Biological Networks Gene Ontology (BiNGO; Maere et al., 2005 Bioinformatics 21:3448–3449) was used, with Bonferroni corrected p-value threshold set at 0.05. Based on enrichment values, the respective GO biological processes were represented in the form of heat map for D, P and DRP stressed transcriptome. Color bar scale in white to red represents enrichment score of 0 to 5. List of all enriched GO biological processes corresponding to down-regulated genes in each stress category is presented in Supplementary File S3.

Supplementary Figure 13

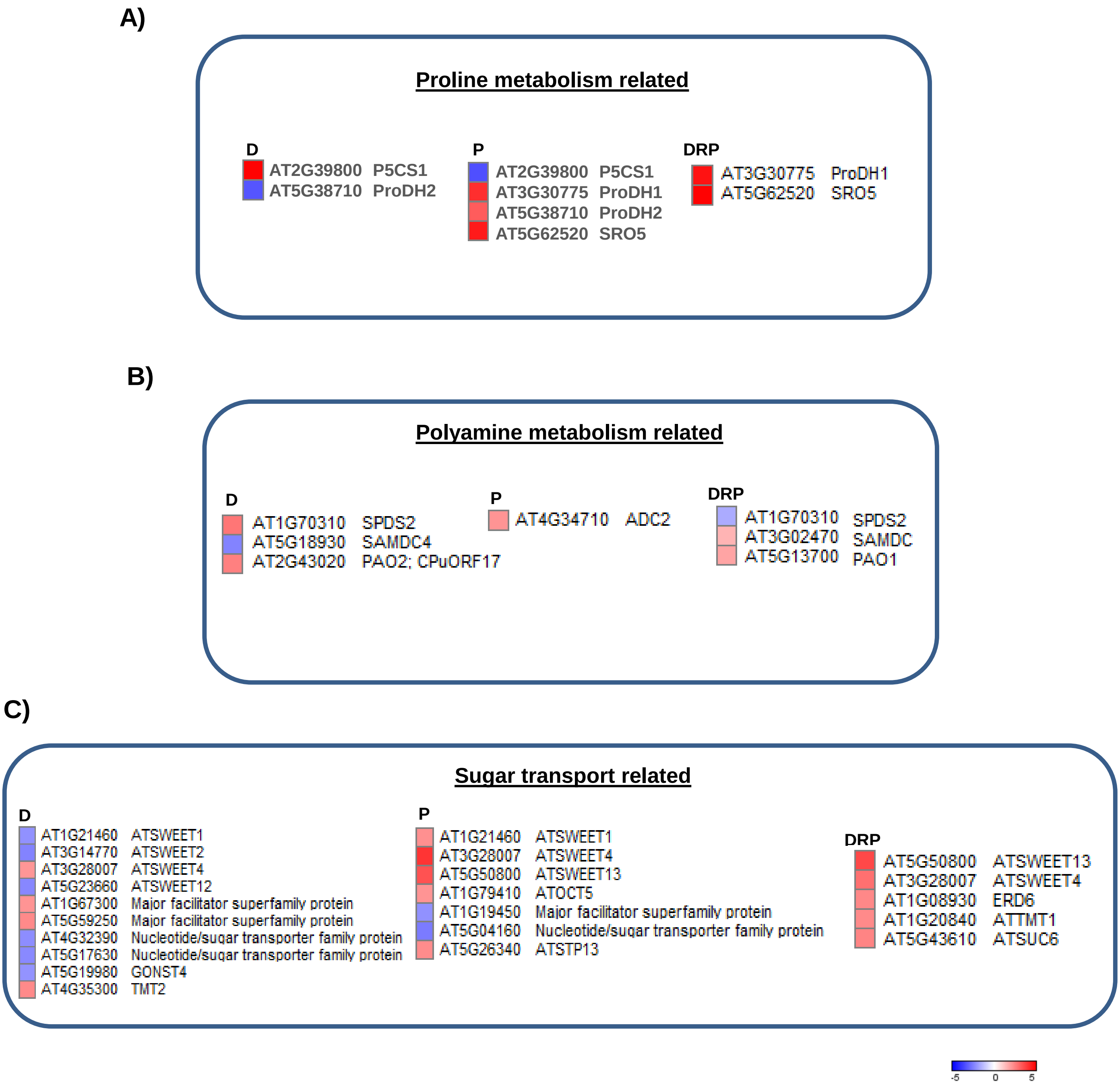

Supplementary Figure 13: Expression profile of genes related to major defense pathways under combined DRP stress. DEGs (fold change cut off >2) under individual D, P stresses and combined DRP treatment were mapped to proline metabolism **(A)**, polyamine metabolism **(B)** and sugar transport **(C)**. The annotation for each pathways was manually retrieved. Fold change values in gene expression (over respective control) were used to plot heat maps where color bar in red and blue represents up- and down-regulated genes respectively.

Supplementary Figure 14

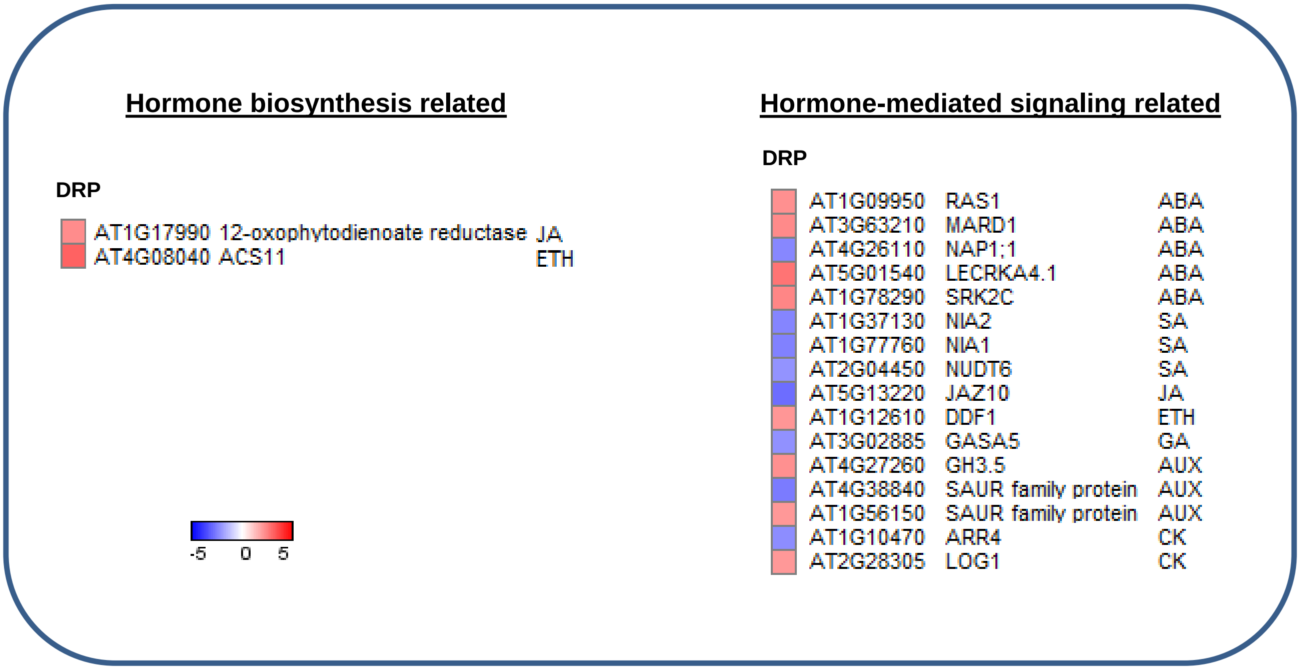

Supplementary Figure 14: Transcriptome profile of genes related to hormone biosynthesis and signaling in DRP treated plants. DEGs (fold change cut off >2) unique to DRP plants (over D and P only stressed plants) were mapped to 'phytohormone' category based on MAPMAN, KEGG annotation and AHD 2.0. Heat maps show expression profile of DEGs corresponding to hormone biosynthesis and signaling under DRP treatment. Fold change values in gene expression (over mock control) were used to plot heat maps where color bar in red and blue represents up- and down-regulated genes respectively. ABA; abscisic acid, SA; salicylic acid, JA; jasmonic acid, ET; ethylene, AUX; auxin, GA; gibberellic acid. Color bar scale shows the fold change range with red and blue color representing up- and down-regulation respectively.

Supplementary Figure 15

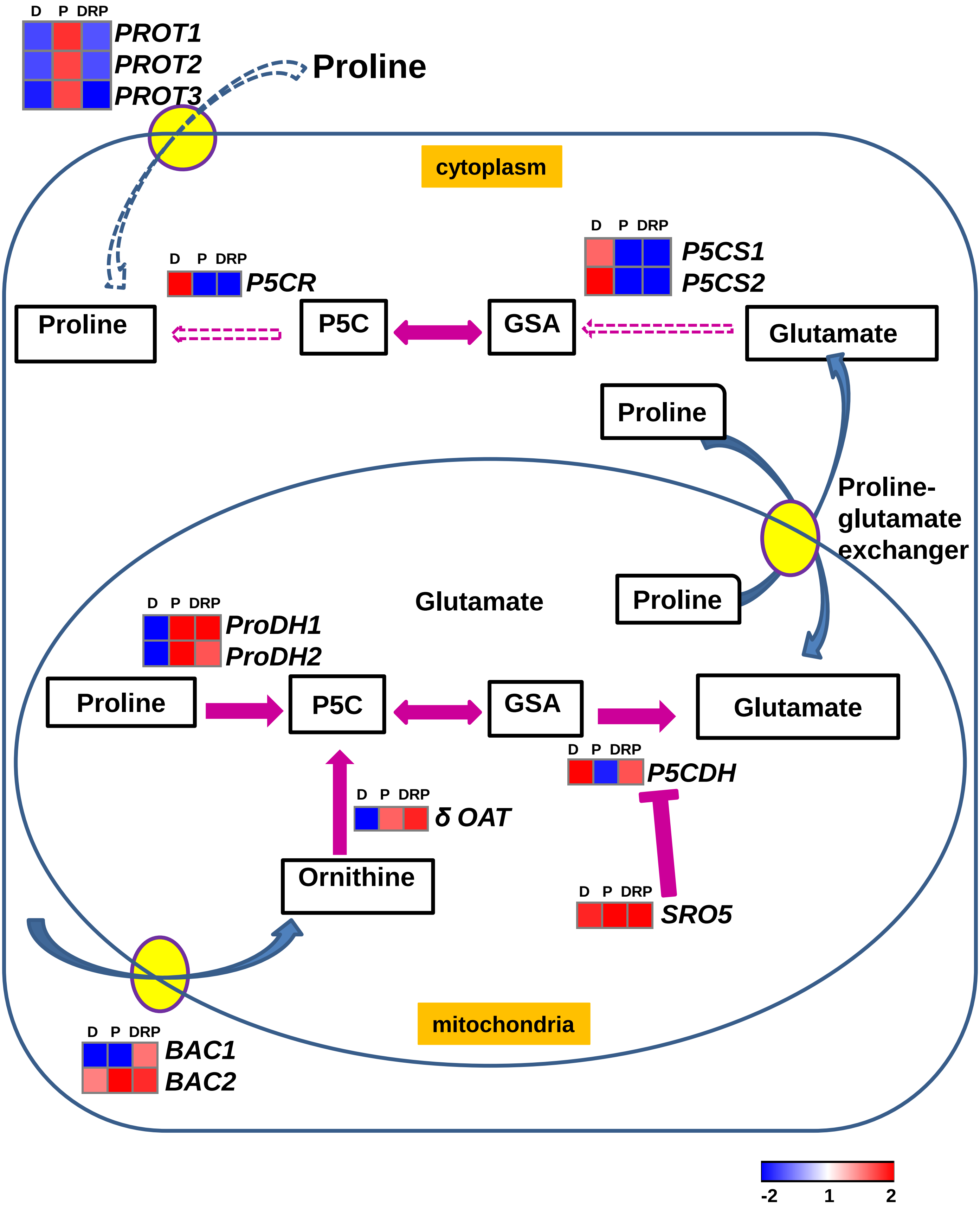

Supplementary Figure 15: Model depicting involvement of proline and P5C metabolism during combined DRP stress. Along every step, expression pattern of the respective genes under D, P and DRP stress is represented in the form of heat map. Color bar scale shows the fold change range with red and blue color representing up- and down-regulation respectively. Looking at the model it can be inferred that under DRP stress P5C tends to accumulate which might drive the observed differential responses over P stress. Colorbar scale in blue to red marks down- and up-regulated genes. P5C; pyrroline-5-carboxylate, P5CDH; pyrroline 5-carboxylate dehydrogenase, ProDH1/2; proline dehydrogenase, δOAT; delta-ornithine amino transferase, P5CS1/2; pyrroline 5- carboxylate synthase, GSA; glutamate semi aldehyde, BAC1/2; basic amino acid career, SRO5; similar to RCD one-5, ProT1/2/3; plasma membrane located transporters. Solid arrows represent up-regulation and broken arrows shows down-regulation in respective metabolic pathways. Henceforth presented outline of proline-P5C pathway is predominantly based on information reviewed in Qamar et al (Front Plant Sci 2015 6: 503. doi: 10.3389/fpls.2015.00503).

Supplementary Figure 16

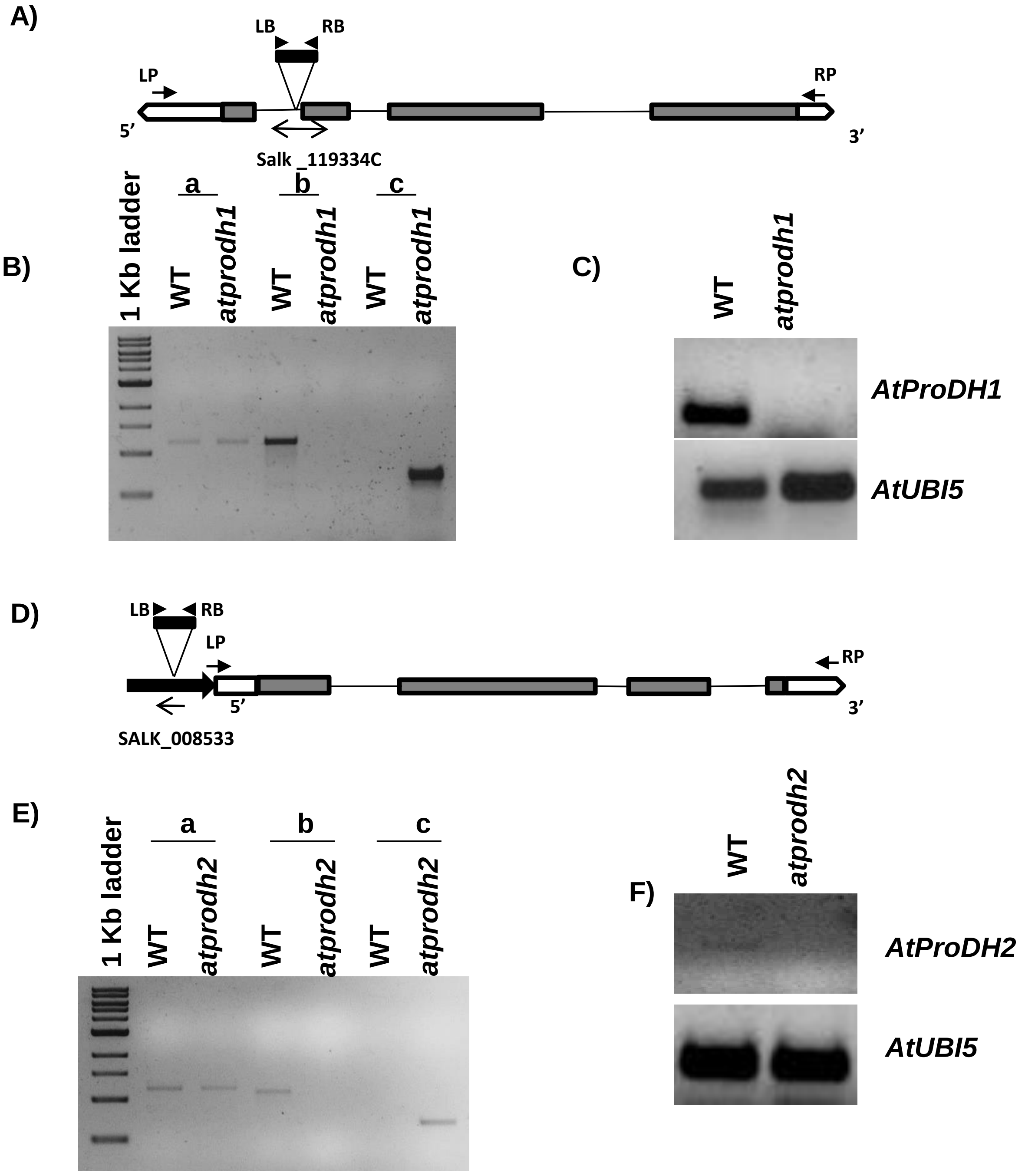

Supplementary Figure 16. Verification of the *A. thaliana* proline dehydrogenase mutants. Maps of T-DNA insertions in *AtProDH1* and *AtProDH2* genes for Salk 119334C and SALK_008533C mutants are shown **(A, D)**. Genotyping of the Arabidopsis T-DNA insertion lines using PCR reactions with **a**; actin gene specific primers show the DNA quality in both wild type and mutant samples, **b**; DNA show a flanking DNA fragment upstream (LP) and downstream of the insertion site (RP) in wild type (WT) but not in mutant, **c**; DNA fragment flanking the T-DNA border (LB) and the downstream of the insertion site (RP) in mutant but not wild type, is shown **(B, E)**. Results depict that both the mutants are homozygous T-DNA insertion lines. Results for semi-quantitative reverse transcriptase PCR reaction showing the expression pattern of target gene in wild-type and mutants under drought recovery are presented **(C, F)**. Since the target gene was not expressive under normal conditions, we used drought-recovery as the conditions inducing their high expression to validate their expression in mutant plants. Semi-quantitative RT-PCR reactions were performed with 50 ng of total RNA for 30 cycles. *AtUBI5* gene expression in control and mutant plants was presented as loading control. The expression level of the target gene was abolished suggesting that the mutants are null mutants. Details of primers used in the genotyping and semi-quantitative RT-PCR are provided in Supplementary Table S1.

Supplementary Figure 17

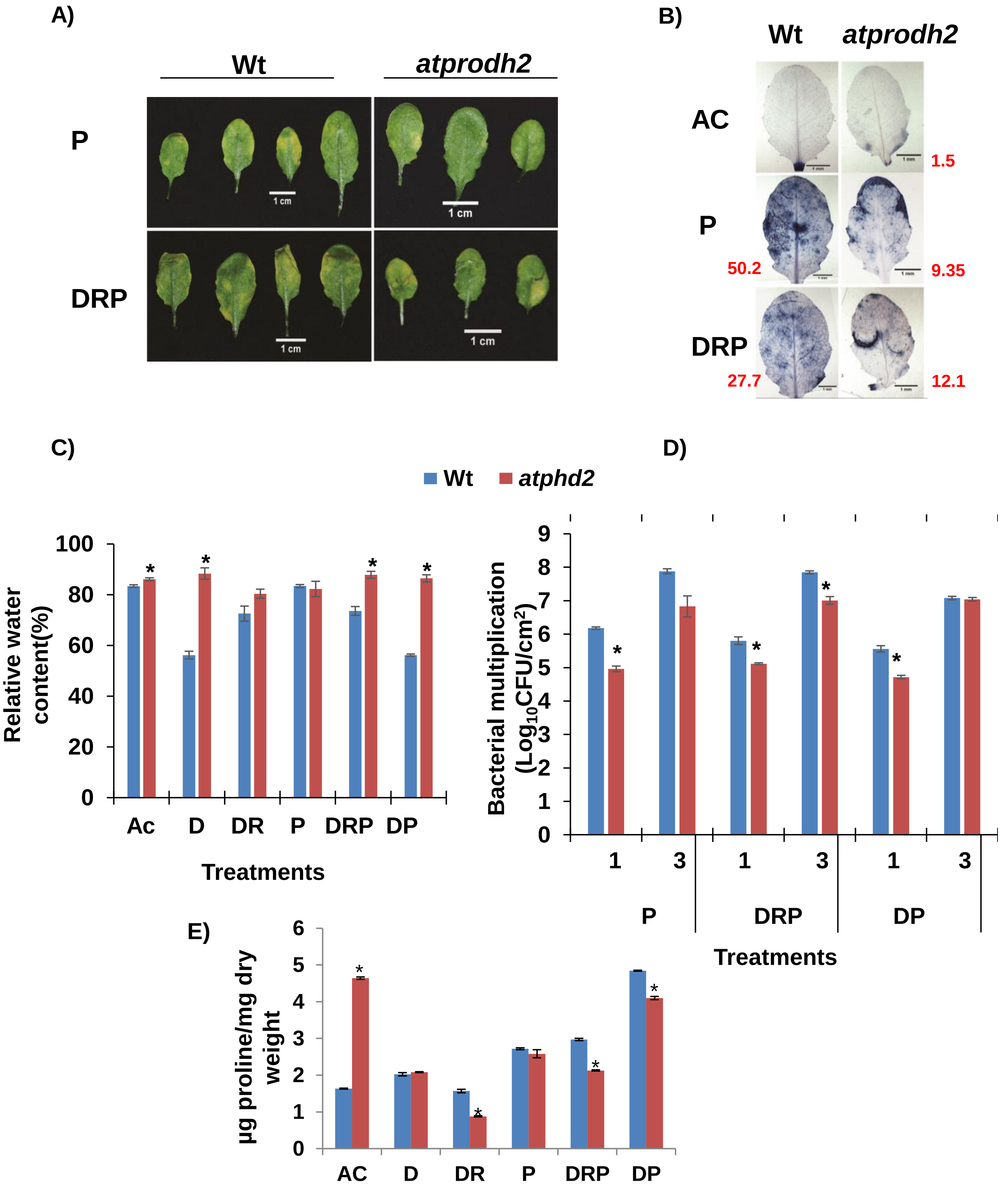

Supplementary Figure 17: Functional analysis of *atprodh2* mutant under individual and combined stress. *atprodh2* mutant and wild type (Wt) plants were exposed to individual drought (D, at 40% FC), pathogen (P, at 10^4 CFU/mL) and drought recovery (DR) and combined drought recovery-pathogen (DRP) and drought-pathogen (DP) treatments. Disease phenotype was captured at 4 days post treatment (dpt) in three biological replicates of Wt and *atprodh2* mutant plants under individual and combined stress treatments **(A)**. Disease associated cell death was captured using trypan blue staining at 3 dpt in two biological replicates of Wt and *atprodh2* plants. Extent of cell death is directly proportional to the intensity of blue color and is represented as fold change over absolute along each image (ImageJ software) **(B)**. Bacterial multiplication in inoculated plants (P, DRP and DP) was monitored at 1 and 3 dpt. Bars represent the mean and SEM for eight biological replicates. Statistical significance for a particular treatment in mutant plant was determined over respective Wt using student's *t*-test. Asterisk represents statistically significant value at $P< 0.05$ **(C)**. Relative water content (RWC) in Wt and mutant plants under different treatments was assessed at 24 hpt. Data represent the mean and SEM for three biological replicates. Asterisk represents statistically significant value at $P < 0.05$ based on Student's *t*-test between well watered plants and other treatments **(D)**. Proline content in Wt and *atprodh2* plants under different treatments was assessed at 24 hpt. Data is the mean of two biological replicates from one experiment. Bars represent mean ± SEM. Students *t*-test was applied to calculate statistical significance in mutant plants over respective Wt treatment. Asterisk represents statistically significant value at $P < 0.05$ **(E)**. AC, absolute control; D, drought; P, pathogen; DR, drought-recovery; DRP, drought-recovery-pathogen; DP, drought-pathogen; WW, well-watered.

Supplementary Figure 18

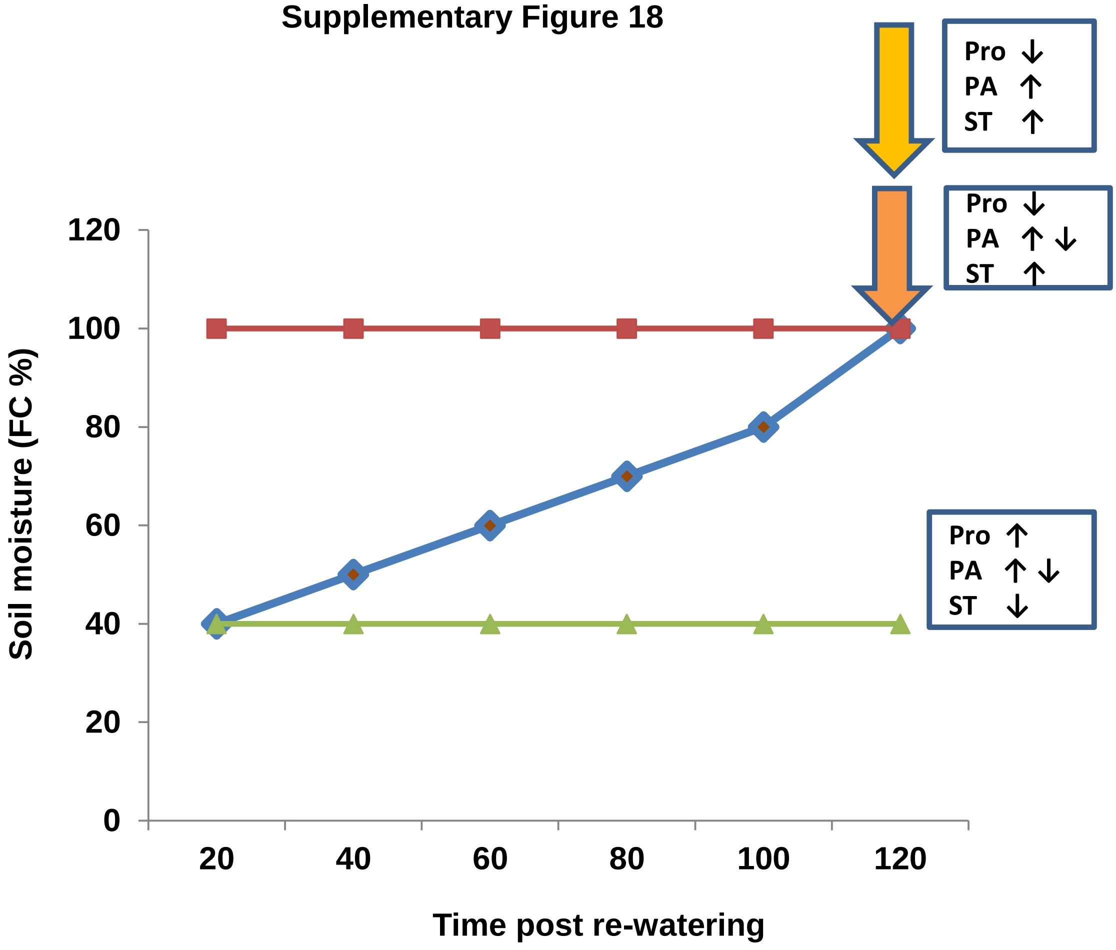

Supplementary Figure 18: Summary of results obtained from the current study on transcriptome profile of different metabolic pathways under combined DRP stress. *A. thaliana* was exposed to individual and combined stress treatments as explained in Supplementary Fig. S1. Microarray hybridization based global transcriptome of leaf samples was carried out and differentially expressed genes under different stress conditions were mapped onto different metabolic pathways. Based on the expression pattern of these genes, alteration in metabolic pathways under each stress conditions were inferred. Red line marks plants maintained at 100% FC, grey line, plants maintained at 40 % FC throughout the experiment. Blue ascending line marks initial drought stress followed by recovery from drought stress and these plants were inoculated with pathogen (green arrows). Pathogen only plants (yellow arrows) were maintained at 100% FC. Pro; proline, PA; polyamines, ST; sugar transport. Downward arrows represent down-regulation and upward arrows shows up-regulation in respective metabolic pathways.
